# Requirements for F-BAR Proteins TOCA-1 and TOCA-2 in Actin Dynamics and Membrane Trafficking during *Caenorhabditis elegans* Oocyte Growth and Embryonic Epidermal Morphogenesis

**DOI:** 10.1371/journal.pgen.1000675

**Published:** 2009-10-02

**Authors:** Chiara Giuliani, Flavia Troglio, Zhiyong Bai, Falshruti B. Patel, Adriana Zucconi, Maria Grazia Malabarba, Andrea Disanza, Theresia B. Stradal, Giuseppe Cassata, Stefano Confalonieri, Jeffrey D. Hardin, Martha C. Soto, Barth D. Grant, Giorgio Scita

**Affiliations:** 1 The FIRC Institute for Molecular Oncology, Milan, Italy; 2 Department of Molecular Biology and Biochemistry, Rutgers University, Piscataway, New Jersey, United States of America; 3 Department of Pathology and Laboratory Medicine, University of Medicine and Dentistry New Jersey—Robert Wood Johnson Medical School, Piscataway, New Jersey, United States of America; 4 University of Milan Medical School, Milan, Italy; 5 Signalling and Motility Group, Helmholtz Centre for Infection Research, Braunschweig, Germany; 6 University of Bonn, Bonn, Germany; 7 Department of Zoology, University of Wisconsin, Madison, Wisconsin, United States of America; University of California San Diego, United States of America

## Abstract

The TOCA family of F-BAR–containing proteins bind to and remodel lipid bilayers via their conserved F-BAR domains, and regulate actin dynamics via their N-Wasp binding SH3 domains. Thus, these proteins are predicted to play a pivotal role in coordinating membrane traffic with actin dynamics during cell migration and tissue morphogenesis. By combining genetic analysis in *Caenorhabditis elegans* with cellular biochemical experiments in mammalian cells, we showed that: i) loss of CeTOCA proteins reduced the efficiency of Clathrin-mediated endocytosis (CME) in oocytes. Genetic interference with CeTOCAs interacting proteins WSP-1 and WVE-1, and other components of the WVE-1 complex, produced a similar effect. Oocyte endocytosis defects correlated well with reduced egg production in these mutants. ii) CeTOCA proteins localize to cell–cell junctions and are required for proper embryonic morphogenesis, to position hypodermal cells and to organize junctional actin and the junction-associated protein AJM-1. iii) Double mutant analysis indicated that the *toca* genes act in the same pathway as the nematode homologue of N-WASP/WASP, *wsp-1*. Furthermore, mammalian TOCA-1 and *C. elegans* CeTOCAs physically associated with N-WASP and WSP-1 directly, or WAVE2 indirectly via ABI-1. Thus, we propose that TOCA proteins control tissues morphogenesis by coordinating Clathrin-dependent membrane trafficking with WAVE and N-WASP–dependent actin-dynamics.

## Author Summary

Cells continuously remodel their shape especially during cell migration, differentiation, and tissues morphogenesis. This occurs through the dynamic reorganization of their plasma membrane and actin cytoskeleton: two processes that must therefore be intimately linked and coordinated. Molecules that sit at the crossroads of membrane remodeling and actin dynamics are predicted to play a pivotal role in coordinating these processes. The TOCA family of proteins represents a case in point. These proteins bind to and deform membranes during processes such as membrane trafficking. They also control actin dynamics through their interactions with actin remodeling factors, such as WASP and WAVEs. Here, we characterize the functional role of TOCA proteins in a model organism, the nematode *Caenorhabditis elegans*. We established that *toca* genes regulate Clathrin-mediated membrane trafficking during oocyte growth. We further discovered that these proteins play an important role in epithelial morphogenesis in developing embryos, and in egg production in adult nematodes. Moreover, the TOCA interacting proteins WASP/WSP-1 and WAVE/WVE-1, as well as other components of the WVE-1 complex, appear to be involved in TOCA-dependent processes. Thus, we propose that TOCA proteins control tissue morphogenesis by coordinating Clathrin-dependent membrane trafficking with WAVE and N-WASP–dependent actin-dynamics.

## Introduction

The coordination and functional cooperation between endocytic trafficking of membranes and membrane proteins with actin-based motility is required for the correct execution of many cellular phenotypes. These include directional cell migration, tissue morphogenesis, cell-fate determination, and the establishment of cell polarity in epithelial and in neuronal cells. Consistently, endocytic trafficking and actin-based motility and dynamics are intimately linked. Results obtained in several species have established that endocytosis and trafficking events rely on propelling forces generated by actin treadmilling [1]. Consistent with these results, an increasing number of actin binding and regulatory proteins have been shown to participate in a variety of internalization and trafficking processes, ultimately controlling the signaling response of cells to extracellular stimuli. In addition, genetic and cellular biochemical evidence has revealed how cycles of endocytosis and recycling of plasma membranes and plasma membrane proteins are essential to promote the spatial restriction of signaling [2]–[5].

However our understanding of the molecular circuitry involved in these processes is still in its early stages. Proteins that sit at the crossroads of membrane remodeling and actin dynamics are predicted to play a prominent role in these processes, simultaneously binding regulators of actin dynamics and sensing or inducing membrane curvature. A prototypical example of this kind of protein is the BAR (Bin, Amphiphysin, Rvs) domain superfamily of proteins—including “classical” BAR domains, F-BAR (FCH-BAR or EFC Extended-FCH) and I-BAR (Inverse-BAR) domains, which have emerged as important players in membrane-remodeling processes [6]. Members of the superfamily are recruited from the cytoplasm to trigger the formation of plasma-membrane extensions, invaginations, tubular organelles, and transport intermediates, including endocytic vesicles [7]–[12]. Much of what is known about the structure-function relationships of the BAR superfamily has been obtained from crystallographic and *in vitro* biochemical studies, showing that members of the family are elongated dimers formed by the antiparallel association of α-helical coiled coils which can deform liposome into tubules of different diameters [13]–[19].

A defining feature of a subfamily of F-BAR-containing proteins that includes three mammalian members, TOCA-1 (Transducer of Cdc42 dependent actin assembly), CIP4 (Cdc42 interacting protein 4) and FBP17 (Formin binding protein 17) (hereafter referred to as the TOCA family), is to possess additional protein:protein interaction domains that enables them to function as signal transducers, physically bridging membrane trafficking with signalling that controls actin dynamics. TOCA-1 and CIP4, through their imperfect HR1/CRIB-like (Cdc42 and Rac interacting binding motif) domain act as direct downstream effectors of the small GTPase Cdc42 [20]–[22]. In addition all members of the TOCA family bind, through their conserved C-terminal SH3 domain, to either prototypical endocytic proteins such as dynamin [6],[8],[12], or to actin nucleation promoting factors (NPFs) such as N-WASP and WASP [12], [22]–[24]. In this latter case, the association of TOCA-1 with the inhibited WASP-WIP complex has been shown to be critical for the activation of Arp2/3-mediated actin polymerization induced by Cdc42 [18],[22],[24].

Despite this wealth of structural and biochemical observations, the functional and cellular roles of the TOCA family proteins have remained largely elusive. Consistent with their biochemical properties, concomitant downregulation of TOCA-1 and FBP17 in vivo resulted in a relative slight inhibition of Transferrin receptor internalization [6],[12],[18]. Whereas the ectopic expression of FBP17 and CIP4, but not of TOCA-1, caused the appearance of membrane tubules, whose accumulation was enhanced by inhibition of dynamin or actin dynamics [6],[8],[12]. Recently, by somatic gene disruption in dorsal epithelial cells, the only *Drosophila* TOCA-family was demonstrated to mediate E-cadherin endocytosis in conjunction with the Cdc42/Par6/aPKC polarity complex [25]. However the precise molecular details in this pathway remain to be elucidated.

Here, by combining genetic approaches in the nematode *C. elegans* with cellular biochemical analysis in mammalian cells we have identified a requirement of the TOCA subfamily of proteins in WASP and WAVE-dependent pathways controlling actin dynamics and membrane trafficking. Specifically we find that CeTOCA-1 and CeTOCA-2 are important for the regulation of Clathrin-mediated endocytic processes during oocyte growth, and in the control of epithelial morphogenesis in developing embryos. Remarkably, mammalian TOCA-1, like *C. elegans* CeTOCA-2, associates with ABI-1, a key member of the WAVE complex. Furthermore mammalian TOCA-1 localizes at tight junction in epithelial cells, suggesting that this function may be conserved.

## Results

### The *C. elegans* TOCA family comprises two genes products, CeTOCA-1 and CeTOCA-2

**Figure 1:**
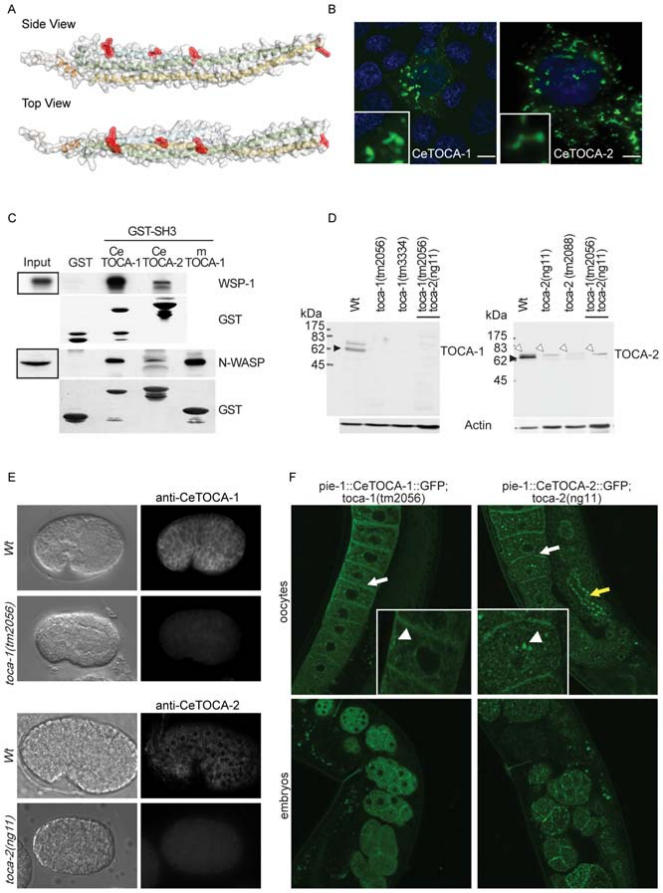
The *C. elegans* TOCA family protein comprises two genes products, *toca-1* and *toca-2*, which display conserved and functional F-BAR and SH3 domains. (A) Model of the predicted tertiary structure of the CeTOCA-1 F-BAR domain. The model was obtained with Phyre software (Protein Homology/analogy Recognition Engine). Residues highlighted in red, corresponding to the ones marked by red asterisks in the sequence alignment shown in Figure S1A, are conserved and involved in phospholipids binding. (B) CeTOCA*s* induces tubular-vesicular structures *in vivo*. Mouse embryo fibroblasts were transfected with the *C. elegans* CeTOCA-1 or 2-fused to GFP. Cells were fixed, stained with DAPI to detect cell nuclei (blue), or processed for epifluorescence. The formation of elongated tubular and vesicular-like structures is shown at higher magnification in the insets. Bar, 10 µm. (C) The SH3 domain of *C. elegans* TOCA-1 is functional. In vitro translated [S35]-M- labeled *C. elegans* WSP-1 (upper panel, WSP-1) or total cellular lysates (1 mg) of HeLa cells were incubated with equal amounts (10 µg) of the SH3 domain of either mammalian or *C. elegans* CeTOCA-1 or CeTOCA-2 fused to GST or GST, as a control. Bound proteins and an aliquot of total cell lysates (50 µg) or of the in vitro translated WSP-1 (1/200 of the material used in the *in vitro* binding experiment) were immunoblotted with the antibodies indicated on the right or exposed to autoradiography to detect in vitro translated WSP-1. The SH3 domain of CeTOCA-1 and CeTOCA-2 migrates slower since both constructs include residues extending 5′, which were required for producing a soluble protein. (D,E) Expression levels of CeTOCA-1 and CeTOCA-2 in Wt and mutant worms. (D) Total cellular lysates of the indicated WT and *toca-1* (left panels) or WT and *toca-2* (right panels) mutant adult worms were immunoblotted with antibodies raised against the C-terminal regions of either *C. elegans* CeTOCA-1 or CeTOCA-2, and against Actin. Black arrows point to TOCA*s* proteins, white arrows indicate unspecific bands. (E) *C. elegans* embryos of Wt or *toca-1(tm2056)* and *toca-2(ng11)* mutants, to show the specificity of the antibodies, were fixed and immunostained with anti-CeTOCA-1 or CeTOCA-2 as indicated (*right*) or processed for differential interference contrast microscopy (DIC) (*left*). Bar, 10 µm. (F) Germline expression of CeTOCA-1 and CeTOCA-2. Full-length CeTOCA-1 C-DNA and full-length genomic CeTOCA-2 were fused to GFP and the transgenes expression was driven by the germ-line specific promoter *pie-1*. Constructs were then bombarded in their respective mutant background (*toca-1(tm2056)* and *toca-2(ng11)*). Images show expression of both CeTOCA-1 (*upper left*) and CeTOCA-2 (*upper right*) in oocytes and in developing embryos (*bottom left* and *right*). Vesicular-like structures in the oocytes are shown at higher magnification in the insets. White arrows point to the plasma membrane in oocytes and white arrowheads to the vesicles. Yellow arrow points to the rachis. Bar 20 µm.

In *C. elegans* two distinct genes display a significant level of overall similarity to the three mammalian members of the TOCA family: CeTOCA-1 and CeTOCA-2. CeTOCA-1 and CeTOCA-2 contain, like their mammalian counterparts, a predicted N-terminal extended FCH or F-BAR domain, a central Cdc42-binding HR1 region, and a C-terminal SH3 domain (Figure S1A). The secondary structure prediction of the N-terminus of the *C. elegans* TOCA-1 protein is in good agreement with the one described for the human F-BAR domain of FBP17 [18] (Figure S1A). Thus we built a structural atomic model of the F-BAR domains of *C. elegans* CeTOCA-1. With an estimated precision of 100% (E-value 2.6 e^−30^), the model predicted this domain to fold into a nearly flat zeppelin shape. This analysis also showed full conservation of all key cationic residues required for membrane lipid bending (Figure 1A), suggesting that the biochemical functions of this domain in CeTOCA-1 is equivalent to that of its mammalian homologues. Consistent with this prediction, the ectopic expression into mammalian cells of GFP-tagged CeTOCA-1 and CeTOCA-2 induced the formation of tubular/vesicular-like structures in nearly 100% of cells expressing the transgene (Figure 1B), like their mammalian homologues [6]. Next, we tested whether the SH3 domain of CeTOCA-1 is also functional and able to associate with one of the known mammalian ligands, N-WASP or *C. elegans* WSP-1. Indeed, immobilized GST-SH3 domains of CeTOCA-1 and CeTOCA-2 bound to mammalian N-WASP and CeWSP-1 (Figure 1C and Figure S1F), CeTOCA-2 SH3 was less efficient than the SH3 domains of human TOCA-1 or CeTOCA-1. Thus, *C. elegans* TOCA*s* appear to possess some of the same biochemical features of their mammalian orthologues.

**Figure 2:**
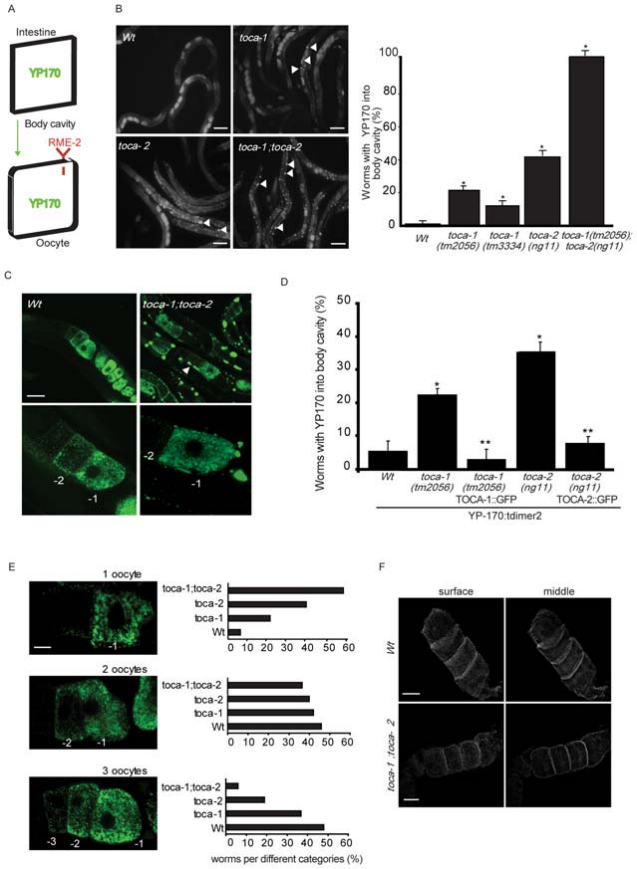
*toca-1* and *toca-2* mutants show impaired endocytosis of the yolk protein YP170 into oocytes. (A–C) Double mutant *toca-1*;*toca-2* worms, expressing YP170 fused to GFP, show accumulation of YP170 in the body cavity. (A) Schematic view of the trafficking of YP170::GFP (YP170, green). The protein is produced in the intestine, secreted in the body cavity, and internalized by the oocytes via a receptor-mediated endocytosis. (B) *Right*, Localization of YP170::GFP in synchronized young adult single and double *toca-1*;*toca-2* mutant worms. Arrows indicate the YP170::GFP impaired accumulation in the body cavity. Bar, 100 µm. *Left*, Quantification of WT and mutant worms accumulating YP170::GFP in the body cavity. Data represent the percentage of worms accumulating YP170 into the body cavity and are expressed as the mean±s.e.m (n = 100) of at least three independent experiments. Asterisks indicate that a significant difference between Wt and mutants was detected (P<0.0001 two-tailed t-test, n = 15). (C) Confocal analysis of YP170::GFP in synchronized at young adult stage Wt and double *toca-1;toca-2* mutant. Lower panels represent magnification of the oocytes filled with YP170::GFP. Oocytes proximal to the spermatheca are numbered as −1. Bar, 100 µm. (D) pie-1::TOCA-1::GFP and pie-1-TOCA-2::GFP rescue the accumulation of YP170::tdimer2 in the body cavity in their respective mutant strains. Quantification of Wt and mutant worms accumulating YP170::tdimer2 in the body cavity. Data represent the percentage of worms accumulating YP170 into the body cavity and are expressed as the mean±s.e.m (n = 100) of at least three independent experiments. One asterisk indicates that a significant difference between Wt and mutants was detected, two asterisks a significant difference between mutants and rescue strains (P<0.0001 two-tailed t-test). (E) *toca-1*;*toca-2* double mutant display reduced endocytosis of YP170 into oocytes. *Left*, examples of the distribution of YP170::GFP in the growing oocytes of Wt worms. Three gonads categories (one, two, or three green oocytes) are shown depending on the accumulation of YP170 into the oocytes. The most represented categories are two (46%) and three (48%) GFP-positive oocytes. *Right*, the distribution of gonad-categories with respect to the indicated genotype is expressed as percentage of the total (n = 100, in three independent experiments) (see also Figure S3B and S3C for quantification). Oocytes proximal to the spermatheca are numbered as −1. Bar, 10 µm. (F) The YP170 RME-2 receptor cellular distribution and expression is not altered in the *toca-1*;*toca-2* double mutant. Confocal images of synchronized WT and *toca-1;toca-2* double mutant worms expressing RME-2::GFP. Images were acquired with Axiovert 200 M microscope using MetaMorph and deconvoluted by AutoDeblur. Surface and middle sections are shown. Note that in the *toca-1*;*toca-2* double mutant, RME-2 is correctly localized at the membrane, like in the WT. We quantified the levels of RME-2 fluorescent signals and show that they are increased on the surface, but significantly reduced in the cell cytoplasms (see Figure S4 for details of the quantification methods). Bar, 10 µm.

To define the functional roles of CeTOCAs in the context of an intact animal model, we generated and analyzed various single and double *toca* deletion mutants in *C. elegans* (described in detail in Figure S1B). All mutations resulted in the elimination of the proteins as shown by immunoblotting with antibodies against the entire C-terminal coiled-coil and SH3 domains of CeTOCA-1 and CeTOCA-2 (Figure 1D). Analysis of CeTOCA-1 and CeTOCA-2 expression by immunofluorescence in developing embryos revealed that the proteins are ubiquitously expressed at various stages (Figure 1E and 1F and Figure S1C and S1D and data not shown). Both proteins labeled intracellular structures and appeared enriched along cell-cell junctions. This was particularly pronounced for CeTOCA-1, which colocalized with the junctional protein AJM-1 (Figure 1E and Figure S1C and Figure S2A), while TOCA-2 appears to be more diffuse in the cytoplasm or cytoplasmic structures, suggesting that these proteins may function in the formation or maintenance of adhesive structures. We further confirmed the intracellular localization of CeTOCA proteins by analyzing the expression of CeTOCA-1::GFP and CeTOCA-2::GFP transgenes in their respective mutant strains lacking the endogenous protein. Attempts to use the endogenous promoters to drive the transgenes failed. We therefore employed the commonly used *pie-1* promoter, to drive maternal protein expression in the germline, and early embryos [26]. This was particularly relevant because of the endocytic defect of oocytes in animals lacking CeTOCA proteins (Figure 2). In oocytes, both gene products are present at the plasma membrane and in vesicular-like structures, consistent with their predicted role in membrane trafficking (Figure 1F). Importantly, we observed a similar staining pattern of endogenous CeTOCA-1 in dissected gonads (Figure S2B) (the anti-CeTOCA-2 antibody was not sufficiently efficient to recognize specifically the endogenous protein in this organ and adult worms). Finally, CeTOCA-2, but not CeTOCA-1 localizes to the partial membranes of the rachis (Figure 1F and Figure S1D), the central core of cytoplasm that connects developing oocytes in the syncytial gonad.

### Efficient Clathrin-mediated endocytosis (CME) of the yolk protein YP170 by oocytes requires CeTOCA proteins

Lipids and proteins derived from yolk are thought to provide essential nutrients required for rapid embryo development. Accordingly, adult *C. elegans* hermaphrodites synthesize massive quantities of yolk particles in their intestines and secrete them basolaterally into the pseudocoelomatic space (body cavity), from which they are taken up into growing oocytes via receptor-mediated endocytosis [27]–[29] (see also Figure 2A). Disruption of CME impedes the internalization of yolk protein YP170, causing characteristic aberrant accumulation of aggregated yolk in the pseudocoelomatic space [29]. Since some of the mammalian TOCA family members are involved in CME [7],[12] we tested whether this was the case also in the nematode.

We observed aberrant accumulation of transgenically expressed yolk marker YP170::GFP [29] in the body cavity of *toca-1(tm2056)*, *toca-1(tm3334)*, and *toca-2(ng11)*, single mutants, as well as *toca-1(tm2056);toca-2(ng11)* double mutant animals. The aberrant distribution of YP170 was most prominent in the *toca-1(tm2056);toca-2(ng11)* double mutant, where nearly 100% of the worms accumulated YP170::GFP in the pseudocoelomatic space (Figure 2B and 2C). Importantly, we could restore the normal distribution of YP170 marker by re-expressing either CeTOCA-1 or CeTOCA-2 specifically in the germline (Figure 2D and Figure S3A), thus demonstrating the requirement for both TOCA gene products in the germ cells.

Furthermore we found that the amount and distribution of YP170 in the oocytes was reduced in toca double mutant, as one would expect from defective internalization. As the growing oocytes move toward the spermatheca, where fertilization takes place, they progressively accumulate YP170, which in Wt distributes in a gradient that generally encompasses three or more oocytes [29]–[30]. Conversely, in endocytosis defective mutants, YP170 is either not detectable, or present only in the last most proximal oocyte (Figure 2E). t*oca-1(tm2056);toca-2(ng11*) strain displays a significant reduction in the number of oocytes positive for YP170, with YP170::GFP only detectable in the single most proximal oocyte, in more than 50% of mutant worms (Figure 2E and and Figure S3B). Additionally, the amount of YP170::GFP, as determined by measuring total fluorescence of the worm with a similar number of YP170-positive oocytes, was significantly reduced (Figure S3C). It is relevant to point out that when we compared worms at a similar stage and displaying a similar number of oocytes in the gonads, most wild type worms had three YP170 positive oocytes (>80% of cases), while most *toca-1(tm2056);toca-2(ng11*) worms had only the most proximal of the oocyte positive for YP170 (>85% of cases), with striking accumulation of YP170 in the pseudocoelomatic cavity (Figure S3B).

Finally, we found that yolk receptor was correctly localized, and actually slightly, but significantly enriched at the plasma membrane, in the *toca-1(tm2056);toca-2(ng11*) double mutant strain (Figure 2F and Figure S4), indicating that the accumulation of YP170 was not due to poor expression or mistargeting of yolk receptors during secretion. Thus, CeTOCA proteins are essential for the efficient endocytosis of yolk protein during oogenesis, suggesting that their primary role is to control CME. The specific role of CeTOCA protein in CME is further demonstrated by the observation that fluid-phase endocytic processes, such as the internalization of ssGFP into coelomocytes was not altered (not shown).

### WSP-1 and the members of the WVE-1 complex are required for yolk uptake

**Figure 3:**
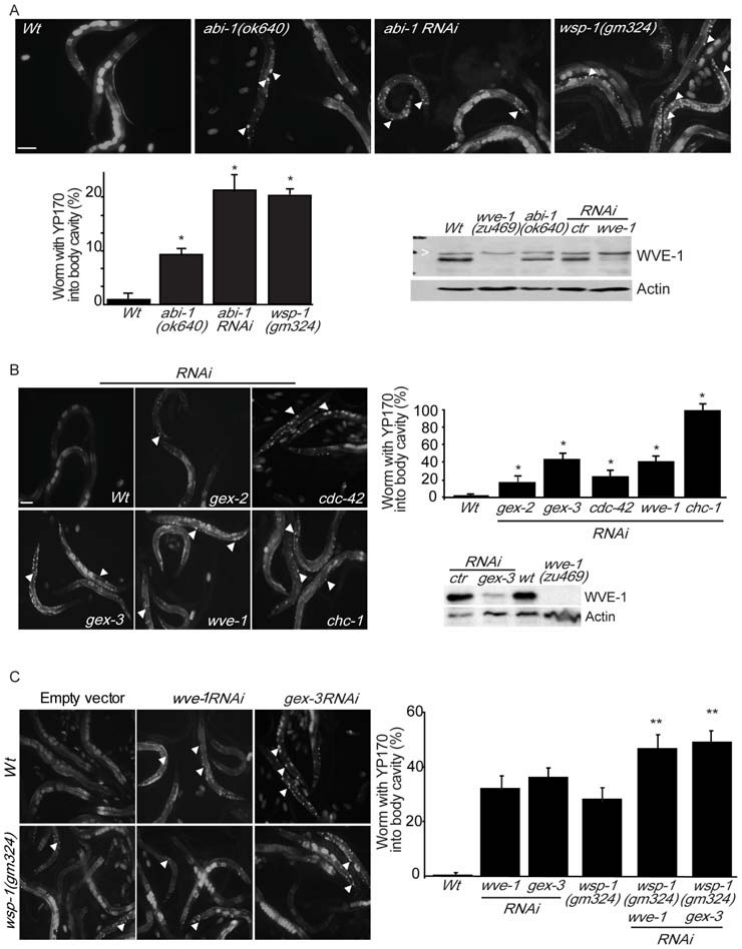
Genetic or functional interference of WSP-1 and of the member of the WVE-1 complex causes accumulation of YP170 into the body cavity. (A) *abi-1(ok640)* or *abi-1(RNAi)* and *wsp-1(gm324)* mutants accumulate YP170 into the body cavity. Localization of YP170::GFP in synchronized young adult Wt, *abi-1(ok640)*, *abi-1(RNAi)*, and *wsp-1(gm324)* mutant worms. Bar, 100 µm. *Bottom graph*, quantification of Wt and mutants worms accumulating YP170::GFP in the body cavity. Data represent the percentage of worms accumulating YP170 into the body cavity and are expressed as the mean±s.e.m (n = 100) of at least three independent experiments. P<0.0001 two-tailed t-test. *Bottom panels*, lysates of adult Wt, *wve-1(zu469)*, *abi-1(ok640)* mutants worms or worms fed with control or *wve-1* specific RNAi-expressing bacteria were immunoblotted with the antibodies indicated on the right. Actin was used as loading control. Note, that in the mutant *abi-1(ok640)*, which retains the binding surfaces to WVE-1, the level of this latter protein is similar to Wt. The asterisk indicates unspecific bands that were occasionally observed depending on the lysis procedure. (B) RNAi-mediated interference of *gex-2*, *gex-3*, or *wve-1* causes accumulation of YP170 in the body cavity. Localization into the intestine, body cavity, and oocytes of YP170::GFP in synchronized young adult worm fed with bacteria expressing the indicated RNAi. *cdc-42* and *chc-1* (Clathrin heavy chain-1) RNAi-treated animals were used as control for defective endocytosis of YP170 [35]. Bar, is 100 µm. *Graph*, quantification of control and RNAi-treated worms accumulating YP170::GFP in the body cavity. Data represent the percentage of worms accumulating YP170 into the body cavity and are expressed as the mean±s.e.m (n = 100) of at least three to four independent experiments. P<0.0001 two-tailed t-test. *Bottom panel*, lysates of control (ctr) and *gex-3* RNAi-treated and of Wt and *wve-1(zu469)* mutant worms were immunoblotted with the antibodies indicated on the right. Actin was used as loading control. (C) RNAi-mediated interference of *wve-1 or gex-3 in wsp-1(gm324)* mutant causes an increased accumulation of YP170 in the body cavity. Localization into the intestine, body cavity, and oocytes of YP170::GFP in synchronized young adult worm fed with bacteria expressing the control and the *wve-1* or *gex-3* RNAi in Wt and *wsp-1(gm324)*. In all images, arrows indicate the YP170::GFP accumulated into the body cavity. *Graph*, quantification of control and RNAi-treated worms accumulating YP170::GFP in the body cavity. Data represent the percentage of worms accumulating YP170 into the body cavity and are expressed as the mean±s.e.m (n = 100) of at least three to four independent experiments. P<0.0001 two-tailed t-test.

The putative role of CeTOCA*s* at the crossroads of membrane trafficking and actin dynamics predicts that this endocytic function may involve TOCA-binding actin regulators. We thus tested whether the two major pathways, mediated by WSP-1 and WVE-1, affect endocytosis. Nematode WVE-1 (also called GEX-1) is associated also in nematode with GEX-2, and GEX-3 [27]–[28], the homologues of mammalian WAVE2, PIR121, NAP-1, and ABI-1 respectively. These proteins together with HSPC300 form a complex [31], which is conserved across different species and is required to activate Arp2/3-dependent actin polymerization [32],[33]. To this end, we analyzed YP170::GFP endocytosis in mutant strains or after RNAi approaches. The RNAi approach was required to assess the role of WVE-1 and its interactors in adult germlines, since complete loss of these proteins leads to embryonic lethality. The accumulation of YP170 in the pseudocoelomatic space was clearly detected in 10% of partial loss of function mutant *abi-1(ok640)*, in 20% of *abi-1(RNAi)* animals, and in 20% of *wsp-1(gm324)* mutant animals [34] (Figure 3A). We observed a similar degree of inhibition of endocytosis after *cdc-42(RNAi)*, while ablation of *chc-1*/Clathrin completely blocked YP170 accumulation by oocytes (Figure 3B) [35].These results suggest that the WSP-1 pathway contributes to optimal endocytosis in the nematode, as it does in mammals (Figure 3A).

Notably, the mutant *abi-1(ok640)* carries a deletion of the exons coding for the C-terminal SH3 domain, which mediates activation of N-WASP in mammals [36]. The N-terminal region of ABI-1, instead, which is essential for the assembly and the stability of the WAVE complex [37]–[39], is predicted to remain unaltered in the *ok640* mutant, suggesting that WVE-1 function might not be disrupted. Consistent with this idea, the level of WVE-1 protein in *abi-1(ok640)* mutant was similar to Wt controls (Figure 3A). WVE-1 levels were significantly reduced, as expected, upon *gex-3(RNAi)* (Figure 3B), another component of the WAVE complex necessary for its stability [40]. Additionally, *abi-1(RNAi)* which is known to destabilize the WAVE2 complex [40], was as effective in inhibiting YP170 internalization as RNAi of *wve-1*, *gex-2*, or *gex-3*, indicating that the WVE-1 complex contributes to endocytosis.

Collectively, these observations support the notion that WSP-1 and, more surprisingly, the WVE-1 complex, which in mammalian cells has never been implicated in CME, are both concomitantly required for efficient internalization of YP170 in *C. elegans* oocytes. In keeping with this notion, RNAi mediated interference of *wve-1* or *gex-3* (Figure 3C) significantly worsened YP170 accumulation into the body cavity of the *wsp-1(gm324)* strain, indicating that the two NPFs act redundantly in this process (Figure 3C).

### CeTOCA-dependent impairment of yolk uptake is accompanied by a reduction in egg production

**Figure 4:**
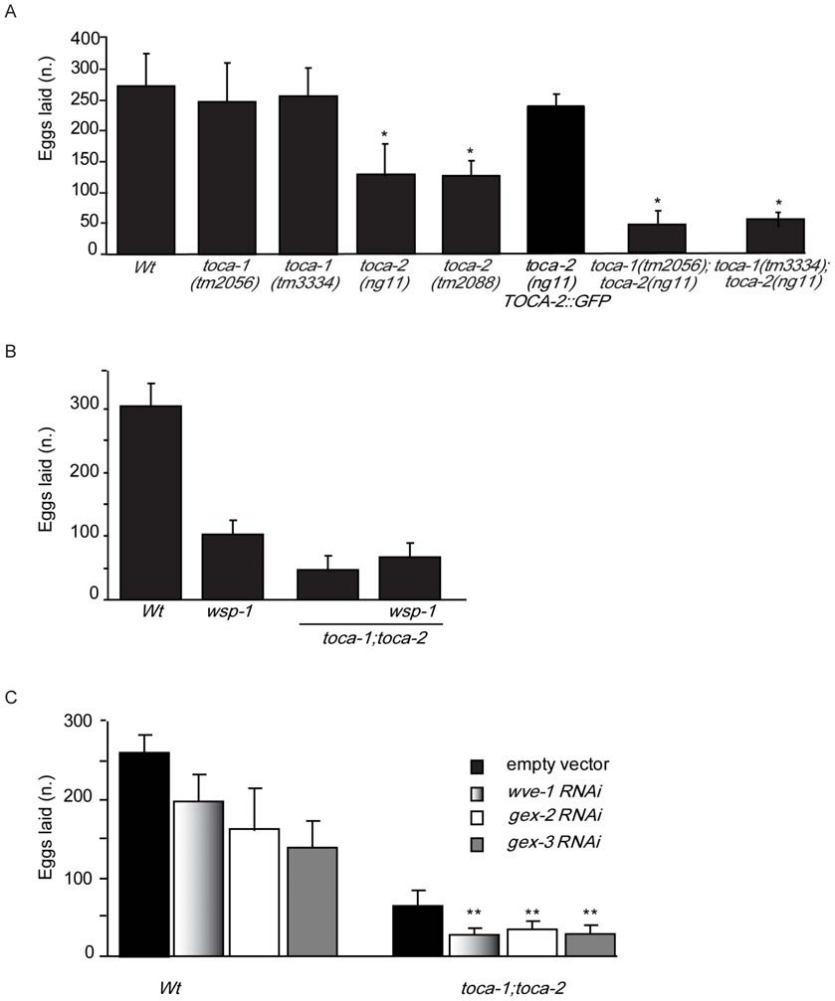
The total number of eggs laid is reduced by mutation in *toca* genes, which genetically interact with *wve-1*, but not with *wsp-1.* (A) *toca-1;toca-2* double mutant worms have reduced number of eggs laid. The total number of embryos, viable and unviable, produced over the lifetime of Wt and mutant hermaphrodites is shown; at least 15 independent hermaphrodites/genotypes were measured. (P<0.0001, two-tailed t-test are indicated by asterisks-mutant vs Wt). Data are expressed as the mean±s.d. (n = 15). (B) Genetic disruption of *wsp-1* does not worsen the impaired eggs production of *toca-1;toca-2* double mutant worms. The total number of embryos, viable and unviable, produced over the lifetime of Wt, single *wsp-1(gm324): wsp-1* double mutant *toca-1(tm2056);toca-2(ng11*): *toca-1;toca-2*, and the triple *wsp-1(gm324);toca-1(tm2056);toca-2(ng11)*: *toca-1;toca-2;wsp-1* hermaphrodites is shown. At least 15 independent hermaphrodites/genotypes were measured. Data are expressed as the mean±s.d. (n = 15). No significant difference between the double *toca-1;toca-2* and the triple *toca-1;toca-2;wsp-1* was detected. (C) RNAi-mediated interference of the members of the WVE-1 complex significantly reduces the number of eggs laid by the *toca-1;toca-2* double mutant. The total number of embryos, viable and unviable, produced over the lifetime of control (empty vector), *wve-1(RNAi) (wve-1* RNAi*)*, *gex-2(RNAi)* (*gex-2* RNAi), and *gex-3(RNAi) (gex-3* RNAi*)* RNAi-treated Wt and *toca-1;toca-2* animals is shown. At least 15 independent hermaphrodites/genotypes were measured. Data are expressed as the mean±s.d. (n = 15). The differences between empty vector and *wve-1*- or *gex-3-*RNAi treated *toca-1:toca-2* double mutant animal were statistically significant (P*<0.009; P**<0.0002, two-tailed t-test). Please note that we did not observe any egg laying (Egl) defects after RNAi of either *wve-1*, *gex-2* or *gex-3*, thus all eggs were laid and the reduction detected is referred to the total number of eggs produced.

Reduced internalization of vitellogenin frequently leads to impaired oocyte production resulting in a reduced total number of eggs laid [29]. Accordingly, while mutation of *toca-1* alleles gave only a slight, but not statistically significant, reduction of the total number of eggs laid, *toca-2(ng11)* and *toca-2(tm2088)* mutant animals laid only 50–60% of the eggs laid by Wt strains and egg production in *toca-1(tm2056);toca-2(ng11)* double mutants was about 20% of Wt (Figure 4A). Germ-line specific expression of CeTOCA-2::GFP in the *toca-2(ng11)* mutant rescued the defect in egg production (Figure 4A), demonstrating the cell autonomy of the requirement for CeTOCA-2.

As previously reported [34], mutation of *wsp-1* caused a significant reduction in the number of eggs laid with respect to Wt worms (Figure 4B). The compound *wsp-1*;*toca-1;toca-2* triple mutant strain, however, did not enhance the phenotype as compared to the *toca-1;toca-2* double mutant (Figure 4B). Conversely, individual RNAi-mediated reduction of either *wve-1* or *gex-2* or *gex-3* caused a somewhat variable (presumably reflecting the variable efficiency of RNAi), but detectable reduction in the number of eggs laid (Figure 4C). Unlike *wsp-1*, the downregulation of *wve-1*, *gex-2* or *gex-3* significantly reduced the number of eggs laid by *toca-1(tm2056);toca-2(ng11*) double mutant worms (from around 70/worm to ∼30) (Figure 4C). Thus, CeTOCA proteins may function redundantly with respect to *wve-1*, but not *wsp-1*, during egg production.

### *toca* double mutant embryos display a Gex (Gut on the exterior) phenotype, an impaired cell surface distribution of AJM-1, and altered organization of actin

**Figure 5:**
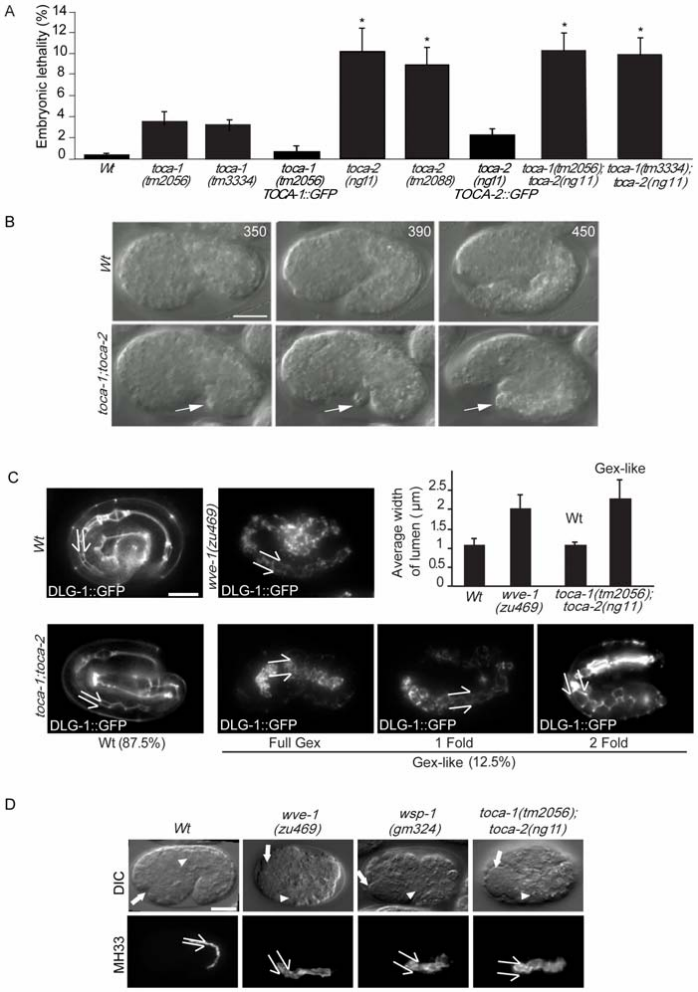
*toca-1* and *toca-2* mutants display embryonic lethality due to a Gex (Gut on the exterior) defect. (A) *toca-1* and *toca-2* mutants display embryonic lethality. Percentage of embryonic lethality of Wt, *toca-1(tm2056)*, *toca-1(tm3334)*, *toca-1(tm2056);pie-1TOCA-1::GFP*, *toca-2(ng11)*, *toca-2(tm2088)*, *toca-2(ng11);pie-1::TOCA-2::GFP*, and compound double *toca-1(tm2056);toca-2(ng11)* and *toca-1(tm3334);toca-2(ng11)*. The percentage of embryonic lethality was determined as described in Materials and Methods by counting the number of un-hatched embryos with respect to the total number of eggs laid by a single worm. Data are the mean±s.e.m (n = 15). P<0.0001, two-tailed t-test is indicated by an asterisk. (B) *toca-1* and *toca-2* mutants display a Gex phenotype. Still images from DIC time-lapse of developing embryos from Wt and *toca-1(tm2056);toca-2(ng11)* double mutant worms. Images were obtained at approximately 350, 390, and 450 min after the first cleavage of embryogenesis. All panels show lateral views of embryos oriented with anterior at left and dorsal up. Arrow points to extruding gut cells (see also Video S1). Bar, 10 µm. (C) *toca-1;toca-2* mutant has similar morphogenesis defects as *wve-1* complex components. *toca-1(tm2056);toca-2(ug11)* mutant shows epithelial morphogenesis defects similar to *gex* mutants. Comparison of morphogenesis in Wt, *wve-1*, and *toca-1;toca-2* double mutant embryos was done using the *dlg-1::gfp* transgene. Double toca mutant display intestinal lumen expansion similar to loss of *wve-1* complex components. Split arrows indicate the lumen width. Percentage of each phenotype is given below representative images (n = 350+). Bar, 10 µm. *Graph*, *toca* mutant with morphogenetic defects (12.5%) die as embryos and show similar degree of lumen expansion as *wve-1* mutant. In contrast, animals that enclose and are viable (87.5%) show lumen width in the Wt range. The width of the lumen was measured using the *dlg-1::gfp* transgene (n = 8+). (D) *toca-1;toca-2* double mutant displays enlarged intestinal lumen like *wve-1* and *wsp-1* mutant. DIC and immunofluorescence images of Wt, *wve-1(zu469)*, *wsp-1(gm324)*, and *toca-1;toca-2* mutant worms. Embryos were fixed and stained with anti-MH33 antibody. White arrows: anterior of the pharynx; white arrowheads: anterior of the intestine. Split arrows indicate the lumen width. Bar, 10 µm.

*toca-1* and *toca-2* single mutants, and *toca-1;toca-2* double mutant, are defective in YP170 oocytes endocytosis and display reduced eggs production, but are capable of moving, mating and appear morphologically normal. Additionally, *toca-1* and *toca-2* single mutants displayed a weakly penetrant, but significant, embryonic lethality (Figure 5A). The embryonic lethality of the double *toca-1(tm2056);toca-2(ng11)* was entirely recapitulated by the single *toca-2(ng11)*, suggesting that loss of CeTOCA-2 is primarily responsible for this phenotype. Identical results were obtained with *toca-1(tm3334);toca-2(ng11)* double mutant (Figure 5A). Importantly, germ line expression of a CeTOCA-2::GFP transgenes in *toca-2(ng11)* strains rescued the embryonic lethal phenotype (Figure 5A), confirming that the lethality results from loss of CeTOCA-2.

In *wve-1/gex-1*, *gex-2* and *gex-3* mutants, dorsal intercalation and migration of ventral and lateral cells are disrupted, resulting in extrusion of intestinal cells concomitant with failure to properly enclose the embryo [41]. We observed a similar set of morphological alterations in *toca-1(tm2056);toca-2(ng11)* double mutant. Time-lapse analysis of developing *toca* double mutant revealed that virtually all dying embryos are defective in morphogenesis (Figure 5B and Video S1). In all these cases gut cells appeared to differentiate normally (Figure 5B). Using the DLG-1::GFP transgene to label the apical junctions [42] we further monitored morphogenesis in live embryos and detected Gex-like morphogenetic defects, including failure of epidermal enclosure (full Gex-phenotype, leading to extrusion of gut to the exterior), 1-fold arrest and 2-fold arrest (Figure 5C, arrow, and Video S1), just as it has been described for loss of the WVE-1/WAVE protein complex. Mutations in WAVE/SCAR components lead to an expansion of the apical lumen of the intestine [40]. Using the DLG-1::GFP transgene, we measured the width of the intestinal lumen and found that *wve-1* mutant and the *toca-1(tm3334);toca-2(ng11)* double mutant display a similar increase in the width of the intestinal lumen (Figure 5C). This Gex-like intestinal morphogenesis defect was further confirmed by staining embryos at the same stage of development with anti-IFB-2 antibody, MH33 (Figure 5D and Figure S5A and S5B), which recognizes intermediates filaments forming the terminal web beneath the microvilli of intestinal cells. WAVE complex mutants and the *toca* double mutant show an expanded MH33 region (Figure 5D and Figure S5B). Together these results strengthen the notion that the embryonic lethality of *toca-1(tm2056);toca-2(ng11)* double or *toca-2(ng11)* mutants is due to a Gex epithelial morphogenesis phenotype [43].

**Figure 6:**
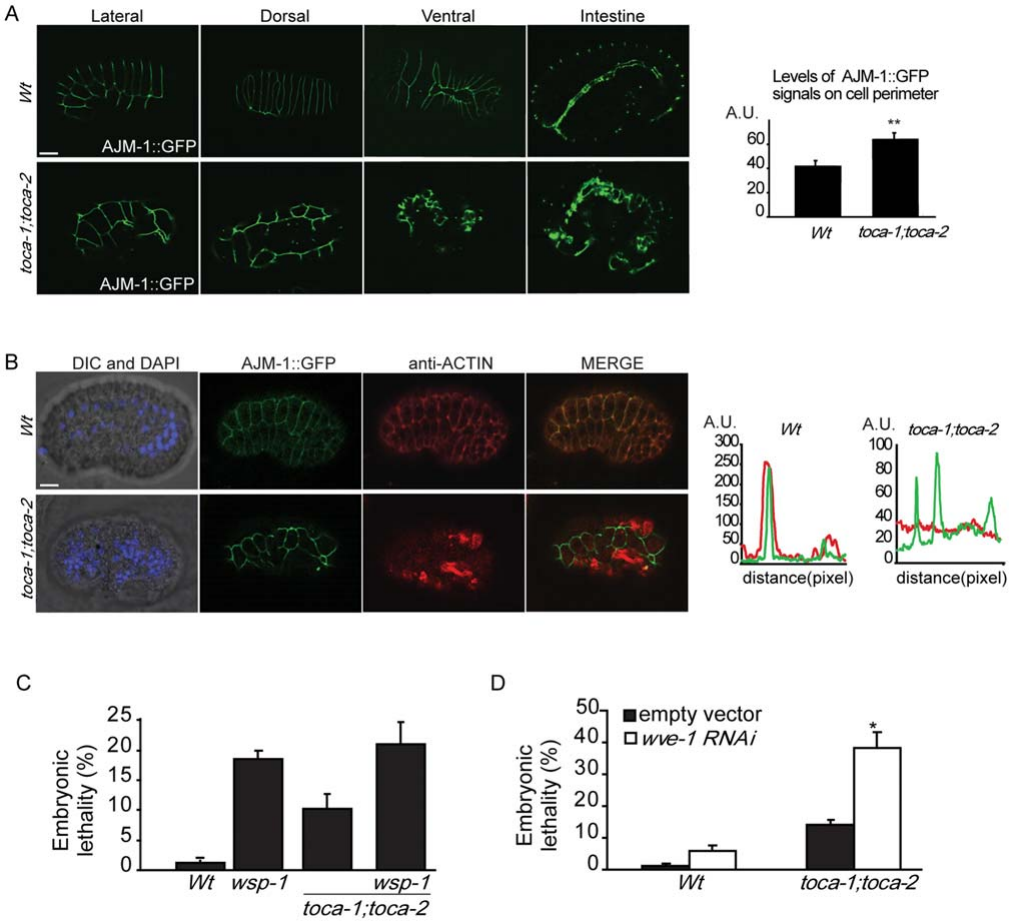
*toca-1* and *toca-2* Gex (Gut on the exterior) embryos display an altered distribution of junctional AJM-1 and disrupted organization of actin. (A) *toca-1;toca-2* double mutant display altered localization of hypodermal cells and increased AJM-1 at cell–cell junction. Representative examples of morphogentic-defective *toca-1;toca-2* double mutant. Lateral, dorsal, ventral, and intestinal view of Wt and *toca-1;toca-2* double mutant expressing AJM-1::GFP. *Right graph*, AJM-1::GFP fluorescent intensity along cell perimeter (arbitrary units, A.U.). Mutants worms with defective hypodermal cell localization at around 360–400 min since the two-cell stage of developments display a 1.5±0.3 (mean±s.e.m., n = 20) fold increase of AJM-1::GFP intensities at cell-junctions. Only hypodermal cells with similar size, shape, and cell perimeter were evaluated. We measured the total intensity of the fluorescent signals along the entire perimeter of hypodermal cells after subtracting background and cytoplasmic signals as described in detail in Figure S6. Several cells (n>20) from different embryos (Wt and *toca-1;toca-2* mutant) were analyzed. (Asterisks indicate P<0.001 two-tailed t-test). Bar, 10 µm. (B) *toca-1;toca-2* double mutant display altered actin organization. Lateral view of WT and *toca-1;toca-2* double mutant embryos expressing AJM-1::GFP after ∼400 min from the first cell division. Embryos were fixed and stained with anti-actin to detect actin or processed for epifluorescence. Bar, 10 µm. AJM-1::GFP and actin intensities (arbitrary units, A.U.) along selected areas (distance, pixel) were quantified by ImageJ software. At least 20 worms displaying Gex phenotypes were analyzed, all displaying similar distribution of actin and of cell surface AJM-1::GFP. (C) Mutation in *toca* genes does not worsen the embryonic lethality of *wsp-1(gm324)*. Percentage of embryonic lethality of Wt, *wsp-1(gm324)*, *toca-1(tm2056);toca-2(ng11)*, and the triple *wsp-1(gm324);toca-1(tm2056);toca-2(ng11)*. The percentage of embryonic lethality was determined by counting the number of un-hatched embryos with respect to the total number of eggs laid by a single worm. At least 15 worms/genotypes were analyzed. Data are the mean±s.e.m (n = 15). No significant difference was detected between the single *wsp-1(gm324)* and the triple *wsp-1(gm324);toca-1(tm2056);toca-2(ng11)* mutant, while *wsp-1(gm324)* and the triple *wsp-1(gm324);toca-1(tm2056);toca-2(ng11)* mutants were significantly different from the double *toca* mutants (P<0.003 two-tailed t-test). (D) *wve-1(RNAi)* enhances the embryonic lethality of *toca-1;toca-2* double mutant animals. Wt and *toca-1;toca-2* double mutant worms were fed with bacteria transformed by control or double-stranded *wve-1* RNA -carrying vectors. Embryonic lethality was determined as described in (C). A significant increase in embryonic lethality (P<0.0001 two-tailed t-test, indicated by asterisk, n = 15) was detected in *wve-1* interfered double *toca* mutant.

We further examined the effect of *toca* mutations on apical junctions, focusing on the key junctional protein AJM-1 (also know as JAM-1) [44]–[45]. In the *toca-1;toca-2* double mutant, AJM-1::GFP was localized at cell-cell junctions, as in the Wt strain, but hypodermal cells failed to intercalate dorsally and to correctly migrate ventrally, similar to *gex-2* and *gex-3* mutants strains [40] (Figure 6A and Figure S5C). Finally, like DLG-1, AJM-1::GFP also marks the apical borders of cells lining the intestinal lumen, which is enlarged in *toca-1;toca-2* double mutants, similar to defects previously reported for *gex* mutants [41]. Furthermore, quantification of AJM-1::GFP at junction (along the cell perimeter) in embryos at similar stage of development revealed a significant 1.5-fold higher fluorescent signal of AJM-1 in *toca-1;toca-2* mutant than in Wt strain (Figure 6A, right graph, and Figure S6), while its overall levels were unchanged as determined by immunoblotting of total worm lysates (not shown). These data suggest an altered cellular trafficking that may lead to increase junctional accumulation of the protein. We caution, however, that junctional AJM-1 levels are an indirect measurement of the amounts on the cell surface of transmembrane proteins, reflecting possible trafficking impairment. Collectively, these results indicate the requirement of CeTOCA proteins in the earliest event of epidermal morphogenesis due to a Gex phenotype and suggest a role in regulation of junctional protein traffic.

Actin also lines the hypodermal cell-cell junctions, where it is required to generate the forces to maintain cell-cell interactions. In the *toca-1;toca-2* double mutant, actin was significantly more diffuse with reduced staining at junctions, suggesting that one of the functions of CeTOCA proteins is to mediate actin dynamics at this site, likely by controlling the recruitment of regulators of actin polymerization (Figure 6B).

WVE-1, the sole *C. elegans* WAVE/SCAR-like protein, and WSP-1, the sole *C. elegans* N-WASP/WASP-like molecule have been shown to act redundantly in regulating actin polymerization during morphogenesis [34],[46]. To test genetically whether the CeTOCA proteins function in the same pathway as WSP-1 and or WVE-1, or in a parallel pathway, we compared the embryonic lethality of single *wsp-1(gm324)* [34] and double *toca-1(tm2056);toca-2(ng11)* with the triple *wsp-1(gm324);toca-1(tm2056);toca-2(ng11)* mutant strain. No enhancement of lethality was observed in strains where both *wsp-1* and *tocas* (∼20% lethality) were mutated, compared to the individual *wsp-1* mutant (Figure 6C), suggesting that the CeTOCAs and WSP-1 function in the same pathway during embryo development. Conversely, when we reduced *wve-1* expression by RNAi interference [since *wve-1(zu469)* mutant is 100% embryonic lethal [40]], we found a significant increase in embryonic lethality [from ∼12% in *wve-1(RNAi)* alone to 40% in the *toca-1(tm2056);toca-2(ng11*) double mutant background] (Figure 6D). Notably, we obtained similar genetic interaction results when we scored the animals specifically for disrupted morphogenesis and localization of intestinal cells by MH33 staining as opposed to failure to hatch (not shown). This latter evidence, combined with the ability of CeTOCAs SH3 domains to bind human N-WASP and *C. elegans* WSP-1, suggests that TOCA*s* might act redundantly with WVE-1, but in the same pathway as WSP-1, presumably in regulating the proper dynamics of actin and its regulators during hypodermal cell migration.

### Mammalian TOCA-1 physically associates with N-WASP and the ABI-1/WAVE2 complex

The interactions in the nematode between CeTOCAs and WSP-1 are consistent with the demonstrated biochemical and cellular role of mammalian TOCA-1 and FPB17, which directly interact with WASP/N-WASP and promote WASP/N-WASP activation at the plasma membrane [22],[24], thus presumably promoting actin dynamics during CME [6],[12]. Consistent with this idea, we found that mammalian TOCA-1 binds to N-WASP and to WIP, an N-WASP interacting regulatory protein, -through the TOCA-1 SH3 domain (Figure 1C and Figure S7A and S7B).

**Figure 7:**
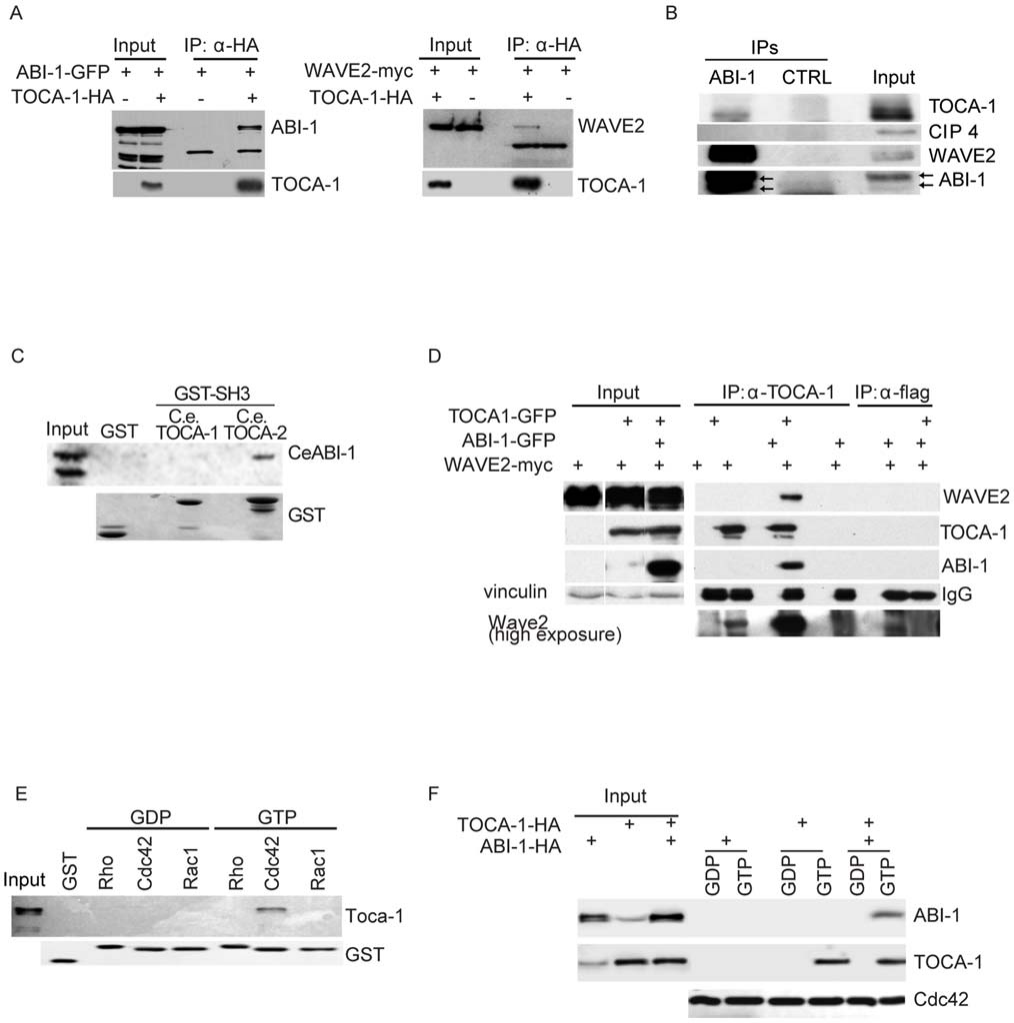
Mammalian TOCA family proteins can bind WAVE2 via ABI-1, in addition to N-WASP. (A) TOCA-1 binds to ABI-1 (left panel) and WAVE2 (right panel). Lysates of HeLa cells transfected with TOCA-1-HA either alone or in the presence of ABI-1-GFP or WAVE2-myc, were immunoprecipitated (IP) with anti-HA antibody. Lysate and IP were immunoblotted with the antibodies indicated on the right. Lower bands in the IP likely represent IgG. (B) Endogenous ABI-1 and TOCA-1 coimmunoprecipitate. Total cellular lysates (2 mg) of HeLa cells were immunoprecipiated with anti-ABI-1 or control, unrelated IgG (CTRL). Lysates and IP were immunoblotted with the indicated abs. Notably, CIP4 does not associate with ABI-1. (C) CeABI-1 interacts with the SH3 domain of CeTOCA-2. In vitro translated, S35-labelled CeABI-1 was incubated with indicated GST fusion proteins (20 µg). Bound proteins and an aliquot of the input (1/50 of the total lysates used in in vitro binding experiment), were revealed by autoradiography. Comassie blue staining of GST-fused proteins is shown at the bottom. (D) TOCA-1 binds to WAVE2 through ABI-1. Lysates of HeLa cells transfected with the indicated combination of plasmids were immunoprecipitated (IP) with anti-TOCA-1 or anti-Flag antibodies (control). Lysates and IP were immunoblotted with the antibodies indicated on the right. The higher exposure blot, on the bottom, shows the presence of endogenous WAVE2 in TOCA-1 IP. (E,F) TOCA-1 binds to active Cdc42 and mediates the formation of Cdc42-TOCA-1-ABI-1 complex. (E) Immobilized GTP-γS (GTP) or GDP-γS (GDP)-loaded small-GTPases fused to GST or GST alone (10 µg) were incubated with total cellular lysates (1 mg) of HeLa cells. Lysates (50 µg), bound materials, and GST-fusion proteins were detected by immunoblotting with antibodies indicated on the right. (F) Lysates (1 mg) from HeLa cells transfected with TOCA-1-HA either alone or in combination with ABI-1-HA were incubated with immobilized GTPγS or GDPγS-loaded GST-Cdc42 (10 µg). Lysates (50 µg), bound materials, and GST-fusion proteins were detected by immunoblotting with antibodies indicated on the right.

The relationship between CeTOCA proteins and the WVE-1/WAVE complex in the nematode is, instead, more complex, suggesting unexpected levels of molecular interactions and regulation. To overcome the lack of reliable antibodies and the intrinsic limitations of cellular biochemistry of *C. elegans*, we set out to define whether any physical or functional interaction between these proteins is conserved in mammals. To this end, we utilized the SH3 domain of TOCA-1 to search for novel interactors, employing phage display libraries of human polypeptides [47]. We found ABI-1 among the most represented interactors (not shown). We validated this interaction by showing that endogenous and ectopically expressed TOCA-1 and ABI-1 coimmunoprecipitated (Figure 7A and 7B), and interacted in *in vitro* binding experiments using immobilized SH3 domain of TOCA-1 (Figure S7C). A similar interaction was also detected between ABI-1 and FBP17 or CIP4 (Figure S7D), indicating that all family members have the capacity to form a complex with ABI-1. Notably, we could recover endogenous TOCA-1, but not CIP4 or FBP17 (not shown), in ABI-1 immunoprecipitates (Figure 7B), suggesting that TOCA-1 is the most likely physiological relevant F-BAR containing interaction partner of ABI-1. In keeping with this notion, we found that *in vitro* translated *C. elegans* ABI-1 readily interacted with the SH3 domain of CeTOCA-2 (Figure 7C), indicating that this interaction is conserved in the nematode.

The quantitatively more relevant binding partner of ABI-1 in mammalian cells is WAVE2 [37]. Consistent with this, we also detected WAVE2 in TOCA-1 and ABI-1 immunoprecipitates (Figure 7B) and in *in vitro* binding experiments using TOCA-1 SH3 domain (Figure S7C). The extent of this interaction was lower than the one between TOCA-1 and ABI-1 (Figure 7A and Figure S7C). These results suggest that ABI-1 may serve as a bridge between TOCA-1 and WAVE2. Accordingly, the amount of WAVE2 in TOCA-1 immunoprecipitates was significantly increased when ABI-1 was over-expressed (Figure 7D).

TOCA-1, like CIP4, was originally identified as an effector of the small GTPase Cdc42. We thus sought to assess whether ABI-1 could be linked directly to Cdc42 in the presence of TOCA-1. The binding of TOCA-1 to purified, GTP-loaded Cdc42 was specific and readily detectable (Figure 7E), as previously reported [22]. More importantly, ABI-1 interacted with GTP-loaded Cdc42 only in the presence of TOCA-1 (Figure 7F). Under these conditions, WAVE2 was also recovered on immobilized and activated Cdc42 (not shown), suggesting that a signaling complex connecting Cdc42-TOCA-1 and ABI-1/WAVE-2 may form. Thus, mammalian TOCA-1 can associate with the WAVE complex through ABI-1, in addition to N-WASP, recapitulating the interactions observed in the nematode.

## Discussion

In this manuscript, we provide evidence that a primary role of the CeTOCA family proteins is to control endocytic processes with important implications for the regulation of actin-dependent epithelial morphogenesis and migration. *toca* genes genetically interact with *wve-1*, acting in the same pathway as *wsp-1*, most likely downstream of *cdc-42*. This, combined with the observation that TOCA family members can form complexes with N-WASP or WAVE2 through ABI-1, indicates that these proteins coordinate N-WASP and WAVE-dependent actin dynamics with membrane trafficking.

### TOCAs, WVE-1, and WSP-1 are essential for optimal Clathrin-dependent endocytosis

The concomitant genetic disruption of the two *C. elegans toca* homologues results in a fully penetrant endocytic defect in oocytes. This defect in yolk uptake likely accounts also for the significant reduction in the number of eggs laid by double *toca-1;toca-2* mutant worms. The finding, however, that CeTOCA-2, but not CeTOCA-1, is enriched at the cortical surface of rachis suggests that this protein may have a specific role beyond vitellogenin endocytosis, possibly by directly affecting oocyte structure and organization. Recently, proteins that display a cortical rachis enrichment, like that of CeTOCA-2, such as Anillin-2 (ANI-2) [48] and Flightless-1 (FLI-1) [49], have been shown to be required for germline morphogenesis and oocyte growth. Both of these proteins are actin-binding factors, pointing to the importance of actin dynamics and architectural organization in this process. In keeping with this observation, one possible, albeit speculative, function for CeTOCA-2 would be to provide a link between actin and membranes during germline morphogenesis. We note however that we did not observe obvious abnormalities in germline structure (e.g. the distribution of nuclei in the syncytium and the size of the oocytes appear relatively normal in *toca-2(ng11)* strain (not shown). More experiments will be needed to assess whether *toca-2* is a germline morphogenesis gene and its precise role in the process.

In YP170 internalization, CeTOCA-1 and CeTOCA-2 act in a redundant fashion. However, in this process, as in embryo development and oocyte growth, CeTOCA-2 display a more prominent role than CeTOCA-1, likely reflecting a differential intracellular localization and/or different binding affinity for their common partners, namely ABI-1 or N-WASP. In these latter cases, the relative low affinities of the interactions observed suggest that CeTOCA*s* may modulate the function of WSP-1 and ABI-1/WVE-1, but are not obligatory partners of either protein complexes.

Notably, the concomitant disruption of *toca-1* and *toca-2* reduced or delayed, but did not abrogate, YP170 entry into oocytes, indicating that these proteins are critical, but not essential for endocytosis of YP170. This is not unexpected given the complexity of CME, where >50 accessory proteins [50],[51], including membrane binding and bending BAR-domain-containing molecules [6],[15], have been shown to aid the core machinery of internalization in a cell context and often cargo-dependent manner [52]. The *C. elegans* genome contains additional F-BAR, N-BAR and SH3-containing proteins. These proteins may, thus, potentially function in a partially redundant fashion with the *tocas* (Figure S8).

The membrane tubulation activity of the F-BAR domain coupled with the ability to bind key actin regulators is predicted to enable TOCA proteins to assist and promote the initial events leading to the internalization of plasma membrane proteins. The genetic evidence provided in this study in part confirms this proposition [7],[12],[18],[19]. We found that: i) the two major actin NPFs WSP-1, and more surprisingly WVE-1 (and its binding partners), are important for optimal internalization of YP170; ii) CeTOCA*s* genetically interact with WVE-1, while acting in the same pathway of WSP-1. The existence of the latter pathway is consistent with the demonstrated role of mammalian TOCA-1 in mediating the biochemical activation of WASP downstream of Cdc42 [22]. According to this model, the direct and concomitant interaction of TOCA-1 and Cdc42 with WASP relieves the autoinhibited state of the latter protein leading to Arp2/3-mediated actin polymerization [22]. A similar mechanisms is likely functional in YP170 internalization, as indicated by the fact that RNAi-interference of Cdc42 phenocopies [35] the removal of WSP-1 and CeTOCA*s* (Figure S8).

The pathways emanating from Cdc42 and regulating endocytosis are, however, more complex that the one depicted here. Indeed, the Cdc42-dependent polarity effector complex, PAR-3/PAR-6/atypicalPKC was also shown to mediate the endocytosis of YP170 in *C. elegans* [35]. Whereas, in *Drosophila*, Cdc42, PAR-6 and atypical PKC, but not PAR-3, have been recently demonstrated to be required for E-cadherin endocytosis, working in concert with CIP4 [25], WASP and Dynamin [53]. The full Cdc42, Par6, Par3, and aPKC complex affect the trafficking of Crumbs and apically applied FM 4–64 in *Drosophila* during neuroectoderm development [54]. While the hierarchical organization and precise mechanisms through which these proteins function remains unclear, these data and our findings suggest a role of a CDC42/PAR-3/atypicalPKC/TOCA/WASP axis in the regulation of early endocytic steps of multiple cargos.

An additional level of complexity is revealed by the requirement for the WAVE/WVE-1 in YP170 endocytosis, suggesting a previously unsuspected regulatory role for the WAVE complex in a *bona fide* Clathrin-dependent internalization process. In mammals, however, no co-localization of this protein complex with Clathrin was ever detected at the plasma membrane, suggesting that it does not directly control internalization [36],[55]. Assuming that this is also the case in the nematode, the most likely role of the WAVE/WVE-1 complex is in later steps of the endocytic route, or to regulate Clathrin-independent pathways.

Finally, it is worth noting that *toca* mutants display more severe endocytic impairment than the combined genetic/functional interference of *wsp-1* and/or *wve-1* or *Cdc42*. This might simply reflect an incomplete down-regulation of *wve-1* and its associated components by RNAi. Alternatively, *toca* genes, by analogy to the role exerted in embryonic development, may act upstream of or in parallel to these NPFs, exerting a modulatory role on their activities, and further regulating additional actin-independent pathways. In favor of this latter possibility, the SH3 domains of all the mammalian TOCA paralogues have been shown to associate with Dynamin [6], a GTPases that is essential for vesicle scission during multiple internalization mechanisms.

One possible scenario that emerges from, and may account for these and previous published observations, is that CeTOCA-1 and CeTOCA-2 may be part of a network of intertwined pathways acting downstream or in parallel to Cdc42 (Figure S8). CeTOCA-1 and CeTOCA-2 may directly connect Cdc42 to WSP-1 and/or Dynamin. Concomitantly, a WVE-1 complex cascade may contribute to the generation of actin dynamics-based forces required for optimal endocytosis. TOCA proteins might drive the correct localization of the WVE-1 complex, rather than modulating its biochemical activity. This possibility is suggested by the interaction between CeTOCA-2 and ABI-1, which is conserved in mammals and nematodes. All of these pathways, including the Cdc42-PAR-6-PAR-3-aPKC complex, may converge, coordinating actin and membrane dynamics during the internalization of different cargos, such as YP170-RME-2 and, presumably, junctional complexes (Figure S8).

### How do TOCAs control epidermal morphogenesis?

Our results indicate that CeTOCA proteins are localized at cell-cell junctions, preferentially at the apical/lateral side, where junctional complexes are also located. A similar localization is also conserved in mammalian epithelial cells. Additionally, at these sites, removal of CeTOCA*s* impairs the organization of cortical filamentous actin and enhances accumulation of the junctional protein AJM-1. Since the primary function of CeTOCA proteins in *C. elegans* appears to regulate endocytosis, it is reasonable to assume that CeTOCA*s* may similarly control the internalization or trafficking of key cell-cell junction proteins. Indeed, intracellular trafficking of junctional proteins is emerging as a critical device to ensure dynamic remodeling of epithelial cell-cell adhesion molecules [56]–[59]. This process may become particularly relevant when epithelial cells migrate, such as during morphogenesis in embryonic development, or in wound closure [60]. Similarly, disruption of CeTOCA proteins may impair the proper internalization of junctional complexes, resulting in altered morphogenetic movements of hypodermal cells. It is of note that defective endocytosis of E-cadherin, a key component of adherens junctions, in the *Drosophila* notum epithelium or dorsal thorax has been recently linked to the only *Drosophila* TOCA family member, CIP4 [25]. In this system, *Drosophila* CIP4 appears to somehow connect the Cdc42-dependent polarity subcomplex, PAR-6/atypicalPKC with WASP and Dynamin, ultimately regulating vesicle scission and internalization of E-cadherin [25],[53]. A similar situation may also occur in hypodermal cells in *C. elegans*. In this case, CeTOCA proteins may directly regulate the activity of WSP-1/WASP, downstream of CDC-42, while cooperating with WVE-1, ultimately mediating the generation of actin dynamics-dependent forces needed for the correct remodeling of junctional complexes during cell migration. Future studies exploiting the mammalian epithelial cell model systems will be required to address these hypotheses. Nevertheless our data suggest that TOCA proteins sit at a critical crossroad of actin and membrane dynamics in the regulation of the intracellular trafficking during epithelial morphogenesis.

## Materials and Methods

### Strains, expression vectors, antibodies, reagents, and cells

The worm strains used are described in Text S1.

GST bacterial expression vectors were generated by recombinant PCR, subcloned in the pGEX-KT vector (Amersham Pharmacia, Piscataway, NJ) and sequence verified. pEGFP-CIP4 and pEGFP-FBP17 were a gift from P. De Camilli. pEGFP-TOCA1 and TOCA1-HA vectors were generated by PCR amplification, subcloned in pcDNA-1-HA or pEFGP vectors and sequence verified. ABI-1HA, WAVE-myc, ABI-1-GFP, Cdc42QL-myc were generated as described before [37],[61]. The antibodies used were: monoclonal anti-CIP4 (BD Transduction Laboratories), anti-Vinculin (Sigma), anti-WIP (Santa Cruz Biotechnology) and monoclonal anti-ABI-1 [37]. The anti-N-WASP antibody was kindly provided by M. Kirschner.

The monoclonal anti-WAVE2 antibody was generated against the C-terminal portion of human protein (VCA domain, aa 422–498) produced as a GST fusion protein.

An anti-CeTOCA-1 rabbit serum and an anti-CeTOCA-2 mouse were generated against a GST-fusion protein containing the CC-SH3 domains (CeTOCA-1 362–592 aa and CeTOCA-2 325–608 aa) of *C.elegans* proteins. A monoclonal anti-mammalian TOCA-1 was raised against amino acids 416–470 fused to GST. Secondary Abs conjugated to Cy3 (Amersham), FITC (Amersham) or Alexa 488 (Molecular Probes) were used. HeLa cells were grown in DMEM (Invitrogen, Carlsbad, CA) supplemented with 10% fetal bovine calf serum (FCS), 100 µg/ml streptomycin, 100 µg/ml penicillin, and 2 mM glutamine. Phoenix and NIH cells were grown in DMEM supplemented with 10% bovine North American serum, 100 µg/ml streptomycin, 100 µg/ml penicillin, and 2 mM glutamine. Transfections were performed using calcium phosphate, FUGENE (Invitrogen), or LipofectAMINE2000 (Invitrogen) reagents, according to manufacturer's instructions.

All genetic experiments and phenotypic analysis in *C. elegans* and cell biochemistry in mammalians are described in Text S1.

## Supporting Information

Figure S1 TOCA genes and proteins. (A) Multiple sequence alignment of TOCA family members from various species (*Homo sapiens*, Hs; *Mus musculus*, Mm; *Xenopus tropicalis*, Xt; *Caenorhabditis elegans*, Ce). Protein sequences were aligned using the ClustalW program. Manual adjustments were introduced on the basis of secondary structure information, and the picture was produced using Jalview. The secondary structure of the F-BAR domain of the human FBP17 (black) and the predicted one of the *C. elegans* TOCA-1 (coloured as in Figure 1A) are reported at the bottom of the alignment. Asterisks indicate residues of FBP17 involved in phospholipids binding. (B) Genomic organization of *toca-1* and *toca-2* genes and of the available deletion alleles. Schematic representation of *C. elegans toca-1* and *toca-2* intron/exon organization (intron = lines; exon = black boxes). The locus position of the putative F-BAR, HR1, and SH3 domains is indicated on top. Bar, 1 Kb. The deletion of the various *tocas* mutant worms utilized is also indicated. The *toca-1(tm2056)* is a deletion encompassing the exon that precedes the one coding for the SH3 domain; *toca-1(tm3334)*, harbours a deletion extending from exon 3 to 4 over the F-BAR domain. Both these deletions result in an out-of-frame shift of the remaining gene products, which cannot be detected by immunoblotting (Figure 1D), indicating that the mutations lead to destabilization of the entire mRNA. *toca-2(tm2088)* is a short deletion of exons 1–4, also causing an out-of-frame shift; finally *toca-2(ng11)*, which was generated by TMP/UV mutagenesis, is a large deletion encompassing almost the entire locus (from exon 4 to 9). To obtain double *toca* mutants, we crossed either one of the two strains carrying the *toca-1* mutated alleles with *toca-2(ng11)*. (C) TOCA-1 and TOCA-2 localization in developing embryos. *C. elegans* embryos were fixed and immuno-stained with anti-CeTOCA-1 or CeTOCA-2 antibodies as indicated (right) or processed for differential interference contrast microscopy (DIC)(left). Bar, 10 µm. (D) TOCA-2 displays a specific localization in rachis. Fluorescent image of pie-1::TOCA-2::GFP showing localization of CeTOCA-2 in Rachis. Arrow points to the plasma membrane. (E) Expression levels of CeTOCA-1 and CeTOCA-2 in Wt and in mutant worms. Total cellular lysates of the indicated Wt and *toca-2* (left panel) or WT and *toca-1* (right panel) mutant adult worms were immunoblotted with antibodies against actin and either CeTOCA-1 or CeTOCA-2, respectively. Arrows point to CeTOCAs proteins. These data indicate the specificity of the anti-CeTOCAs ab. (F) The SH3 domains of CeTOCA-1 and CeTOCA-2 bind mammalian N-WASP. Total cellular lysates (1 mg) of HeLa cells were incubated with different amounts (5 or 15 µg, respectively) of the SH3 domain of CeTOCA-1 or CeTOCA-2-fused to GST or GST, as a control. Bound proteins and an aliquot of total cell lysates (100 µg) were immunoblotted with the antibodies indicated on the right.
(3.04 MB TIF)

Figure S2 Toca localization at junction and in germline. (A) CeTOCA1 and AJM-1 partially colocalize at cell-cell junction. Confocal lateral view of Wt embryos expressing AJM-1::GFP at 1.5 fold stage. Embryos were fixed and stained with anti-CeTOCA-1 or processed for epifluorescence. Bar, 10 µm. (B) Germline and oocytes expression of CeTOCA-1. Germline and oocytes (surface and middle view) from Wt animal showing CeTOCA-1 expression. Gonads were dissected, fixed, and stained with anti-CeTOCA-1. Bar, 20 µm. Images were acquired with Axiovert 200 M microscope using MetaMorph and deconvoluted by AutoDeblur.
(5.08 MB TIF)

Figure S3 OCA proteins in yolk endocytosis. (A) pie-1::TOCA-1::GFP and pie-1::TOCA-2::GFP rescue the YP-170::tdimer2 accumulation in the body cavity of *toca-1(tm2056)* and *toca-2(ng11)* mutants. Localization of YP170::tdimer2 in synchronized young adult single *toca-1* and *toca-2* mutant worms and in pie-1::TOCA-1::GFP and pie-1::TOCA-2::GFP lines in their respective mutant background. Arrows indicate examples of YP-170::tdimer2 accumulation into the body cavity. Bar, 100 µm. (B) Double mutant *toca-1;toca-2* display reduced YP-170::GFP endocytosis in the oocytes. Examples of the most represented categories of GFP-positive oocytes in Wt (3 oocytes, 80%) and *toca-1;toca-2* mutant (1 oocyte, >85%) when comparing animals with the same number of oocytes in the gonad (see DIC images). The numbers −1, −2, −3, and −4 indicate the GFP positive oocytes from the more proximal to the more distal. (C) Double *toca-1;toca-2* mutant has reduced YP-170::GFP in the oocytes. *Left*, quantification of YP-170::GFP into oocytes comparing Wt and *toca-1;toca-2* with the same gonad category (3 GFP-positive oocytes). The numbers −1, −2, and −3 indicate the GFP positive oocytes from the more proximal to the more distal. YP-170::GFP fluorescent intensities (arbitrary units, A.U.) along selected (distance, pixel) area were quantified by ImageJ software (see Materials and Methods). Different areas within the three oocytes (e.g., yellow square) from at least 20 animals were analyzed. *Right*, graph showing the average GFP intensity per oocytes (*left*) or the overall GFP intensity (*right*) in Wt and *toca-1;toca-2* mutant. Asterisks indicate P<0.0001 by two-tailed t-test.
(2.93 MB TIF)

Figure S4 RME-2 levels in *toca-1;toca-2* oocytes. RME-2, the yolk receptor, is correctly localized and enriched at the plasma membrane. RME-2::GFP fluorescent intensities (arbitrary units, A.U.) along selected (distance, pixel) areas and lines were quantified by ImageJ software (see Materials and Methods). Different areas from at least 20 Wt and *toca-1;toca-2* animals were analyzed. The images in red represent a typical example of Wt and *toca-1;toca-2* animals and were obtained by applying a threshold algorithm (ImageJ) to equalize and remove background staining and evidence pixel intensities values above threshold, which correspond to surface RME-2 signals. This procedure permits us to appreciate that the levels of cortical RME-2 are higher in *toca-1;toca-2* animals with respect to Wt. *Graph*, the GFP intensity along the junctions (*upper*) and the average intensity of cytoplasmic RME-2 per area (*bottom*) is plotted for Wt and *toca-1;toca-2*.
(1.68 MB TIF)

Figure S5 Intestinal morphology defects of *toca-1;toca-2*. *toca-1* and *toca-2* mutants display a Gex phenotype. (A) *toca-1;toca-2* double mutant worm displays altered intestinal morphology during embryo development. Wt and the indicated mutant worms were fixed and stained with anti-MH33 antibody or DAPI to detect the intestinal cells and cell nuclei, respectively. Embryos die at 1.5 fold stage, just before elongation starts; DAPI shows that Wt and mutants have a similar number of nuclei, indicating a similar developmental stage. Bar, 10 µm. The percentage of gut-defective embryos of the various genotypes, quantified as described in Materials and Methods, is shown in the bottom graph. Please note that in the case of *gex-3(zu196)* we used a balanced heterozygous strain *OX169 gex-3(zu196)/DnT1* in which only 25% of the progeny is homozygous for *gex-3(zu196)* according to Mendelian distribution. Nearly 100% of these homozygous *gex-3(zu196)* embryos display the morphogenetic intestinal defect as previously reported (Soto et al., 2002). Data are the mean±s.e.m. (n = 100) of at least three independent experiments. P<0.0001, two-tailed t-test is indicated by an asterisk. (B) Intestinal morphology of Wt and mutant embryo at different stages of development. Wt and *toca-1;toca-2* mutant worms were fixed and stained with anti-MH33 antibody. All dying (∼12% of total embryos, Figure 5A) *toca-1;toca-2* embryos are arrested at 1.5 fold stage, just before elongation starts (*left*); A significant fraction of *toca-1;toca-2* embryos display altered intestinal morphology with enlargement of the intestinal lumen at the 2 fold stage with respect to Wt. Of note, at this stage it is easy to appreciate that MH33 display an apical distribution as previously reported (Patel et al., 2008) (*right*). Bar, 10 µm. (C) *toca-1;toca-2* Gex (Gut on the exterior) embryos display an altered epidermal cell morphology typical of gex mutants. *Left*, lateral and ventral view of Wt and *toca-1;toca-2* expressing AJM-1::GFP. *Right*, a scheme of the morphogenetic defects caused by loss of *toca-1* and *toca-2*. Loss of *tocas* leads to the distinctive Gex (Gut on the exterior) phenotype due to failures in cell movement and cell shape changes. Lateral view: *gex* embryos fail to initiate epidermal ventral movements. By 400 min after first cleavage, Wt embryos initiate circumferential constrictions to squeeze the embryo into a worm. *gex* embryos undergo constriction that leads the epidermis to collapse inwardly. The internal organs (pharynx and intestine) end morphogenesis exposed on the ventral surface. Ventral view: in *gex* embryos epidermal cells fails to correctly migrate to the ventral side as in the Wt. Arrows indicate epidermal cells. Bar, 10 µm.
(2.88 MB TIF)

Figure S6 The surface levels of AJM-1 of *toca-1;toca-2* mutant embryos are slightly higher than wt embryos. Quantification of AJM-1 along the cell perimeter of hypodermal cells in WT and *toca-1;toca2* mutant embryos. Embryos at the two-cell stage were kept at 22° for 5 hours before fixation between 360 and 400 min, as indicated, when the “bean shape stage” was reached. Embryos were stained with anti-actin (not shown). The second and third raw images were used to determine the levels of F-actin at junctions as described in Figure 6B, or processed for epifluorescence or DIC. Images were captured with a Leica Microsystems confocal microscope using the HCX PL APO CS 63.0×1.40 OIL objective lens, and objective zoom (3.49×). Exposure time and gain setting were fixed as follows: (Dapi PMT1 (Photo Multiplier Tube) = 500, GFP-PMT2 = 583, Cy3-PMT3 = 550, PMT Trans (HV) = 298). Identical settings were used for all samples so that direct comparison of the signal intensities among the images of embryos of different genetic backgrounds was possible. The ImageJ threshold alogorithm (red channels) were then applied to eliminate cytoplasmic and background signals by placing “Regions of Interest” (ROI) over areas outside the junctional contour. We manually determined the cell perimeters and calculated mean intensities and perimeter length. The mean intensitiy values were then multiplied by the perimeter of each cell to obtain the total intensity along the cell perimeter, as exemplified in the boxed cells on the bottom raw (the line around the cells is meant to outline the chosen cell, not the actual perimeter). Examples of the values obtained for the outlined (white circles) cells are shown. We repeated the procedures to obtain statistically meaningful data that are expressed as mean±s.e.m (n>20 from at least 4 to 5 independent embryos of each genotype). The values of the total intensities along the cell perimeters were plotted as described in Figure 6A. Bar is 10 µm. The results indicate that there is a slight, but significant enrichment of AJM-1 at the junction. This may suggest an altered trafficking of junctional proteins. However, AJM-1 is not a transmembrane protein in *C. elegans*, but rather associates with apical junctional molecules and thus presumably reflects the cell surface levels of such transmembrane proteins. P<0.0002, two-tailed t-test is indicated by asterisks.
(1.75 MB TIF)

Figure S7 Biochemical interactions of Toca-1 protein in mammalian cells. (A,B) The SH3 domain of mammalian TOCA-1 binds to N-WASP and WIP. (A) Purified N-WASP was incubated with the indicated SH3-GST fusion proteins (10 µg). Input (1/10 of the total) and bound N-WASP and GST fusion proteins were detected with the antibodies indicated on the right. (B) Lysates (1 mg) from HeLa cells were incubated with the indicated SH3-GST fusion proteins (10 µg). Lysates (50 µg), bound material, and GST proteins were detected by immunoblotting with the indicated antibodies. (C) The SH3 domain of mammalian TOCA-1 binds to ABI1 and WAVE2. Lysates (2 mg) of HeLa cells were incubated with the SH3 domain indicated (SH3-GST) or GST alone as control (GST). Lysates (100 µg) and bound proteins were immunoblotted with the indicated antibodies. (D) CIP4 and FBP17 interact with ABI1. Lysates of HeLa cells expressing ABI1-HA alone or in combination with CIP4-GFP or FBP17-GFP were immunoprecipitated (IP) with anti-ABI1 antibody. Lysate and IP were immunoblotted with antibodies indicated on the right.
(1.31 MB TIF)

Figure S8 Working model of TOCA proteins signalling network in the regulation of membrane trafficking. Working model of TOCA proteins signalling network in the regulation of membrane trafficking. TOCA-1 and TOCA-2 may link nucleating promoting factors to the plasma membrane (not shown) via their F-BAR domain and integrate signalling pathways controlled by the small GTPases Cdc42. A Cdc42/WASP(WSP-1)/TOCA-1/2, in analogy to that demonstrated in mammalian (Itoh et al., 2005; Tsujita et al., 2006), may directly promote localized actin dynamics during early steps of Clathrin-mediated endocytosis (CME). The polarity complex PAR-3/PAR-6 whose activity is required for endocytic and recycling events downstream of Cdc42 (Balklava et al., 2007) may define an alternative branch of the pathway that may also converge in controlling the TOCA-1/2/WASP(WSP-1) axis. An unexpected contribution of the WAVE(WVE-1) axis in this process is evidenced by; i) the increased accumulation of YP170 after interference with WAVE (WVE-1) complex components; ii) the genetic interactions of these latter genes with *toca-1/2*; iii) the biochemical link between TOCA-1/2 and ABI1, which is conserved also in mammals. The WAVE (WVE-1) complex may function in later endocytic steps of CME since, at least, in mammals it does not localize to Clathrin-coats at the plasma Membrane (Benesch et al., 2005). The precise relation of the WAVE (WVE-1) complex with CDC42/TOCA-1/2 is unclear at present. However, TOCA-2 appears dominant with respect to TOCA-1. TOCA1/2 may also directly associate to Dynamin (Itoh et al., 2005; Tsujita et al., 2006), whose pinching activity is critical to promote vesicle scission. Dynamin- and actin-dependent activities may work in concert with TOCA-1/2 to promote tubule scission. Other F-BAR containing proteins, such as Nostrin may add further layers of complexity to this network, which may coordinate membrane tubulation and curvature sensing with the activity (WASP/WSP-1) (Itoh et al., 2005; Tsujita et al., 2006). Green lines indicate potential genetic interactions; black line indicates genetic and/or biochemical interactions.
(0.16 MB TIF)

Text S1 Supplementary Materials and Methods.
(0.04 MB DOC)

Video S1 *toca-1;toca-2* double mutants show a Gex phenotype. Time-lapse observation of embryogenesis with Nomarski microscopy of Wt and *toca-1;toca-2* double mutant worms. Movie recording started approximately 300 min after first cleavage. Frames (451) were taken every 1 min for a total of 7 1/2 hours.
(1.78 MB AVI)
