# Supplementary figures and images for: Requirements for F-BAR Proteins TOCA-1 and TOCA-2 in Actin Dynamics and Membrane Trafficking during Caenorhabditis elegans Oocyte Growth and Embryonic Epidermal Morphogenesis
Source: PLoS Genet. 2009 Oct 2;5(10):e1000675. doi: 10.1371/journal.pgen.1000675 (PMC2744924; doi:10.1371/journal.pgen.1000675)

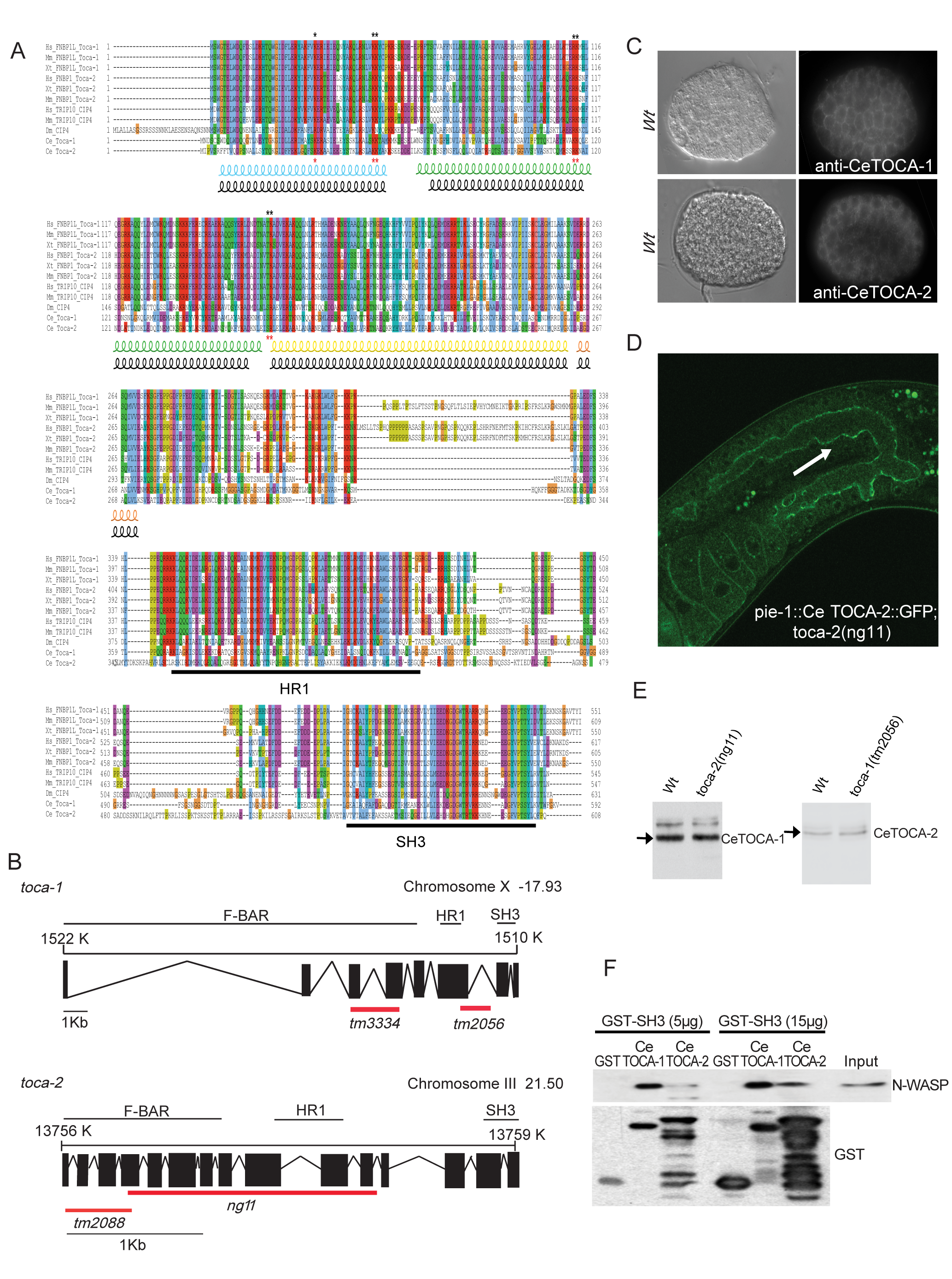

Supplement: Figure S1 — TOCA genes and proteins. (A) Multiple sequence alignment of TOCA family members from various species (Homo sapiens, Hs; Mus musculus, Mm; Xenopus tropicalis, Xt; Caenorhabditis elegans, Ce). Protein sequences were aligned using the ClustalW program. Manual adjustments were introduced on the basis of secondary structure information, and the picture was produced using Jalview. The secondary structure of the F-BAR domain of the human FBP17 (black) and the predicted one of the C. elegans TOCA-1 (coloured as in Figure 1A) are reported at the bottom of the alignment. Asterisks indicate residues of FBP17 involved in phospholipids binding. (B) Genomic organization of toca-1 and toca-2 genes and of the available deletion alleles. Schematic representation of C. elegans toca-1 and toca-2 intron/exon organization (intron = lines; exon = black boxes). The locus position of the putative F-BAR, HR1, and SH3 domains is indicated on top. Bar, 1 Kb. The deletion of the various tocas mutant worms utilized is also indicated. The toca-1(tm2056) is a deletion encompassing the exon that precedes the one coding for the SH3 domain; toca-1(tm3334), harbours a deletion extending from exon 3 to 4 over the F-BAR domain. Both these deletions result in an out-of-frame shift of the remaining gene products, which cannot be detected by immunoblotting (Figure 1D), indicating that the mutations lead to destabilization of the entire mRNA. toca-2(tm2088) is a short deletion of exons 1–4, also causing an out-of-frame shift; finally toca-2(ng11), which was generated by TMP/UV mutagenesis, is a large deletion encompassing almost the entire locus (from exon 4 to 9). To obtain double toca mutants, we crossed either one of the two strains carrying the toca-1 mutated alleles with toca-2(ng11). (C) TOCA-1 and TOCA-2 localization in developing embryos. C. elegans embryos were fixed and immuno-stained with anti-CeTOCA-1 or CeTOCA-2 antibodies as indicated (right) or processed for differential interference contras [file pgen.1000675.s001.tif]

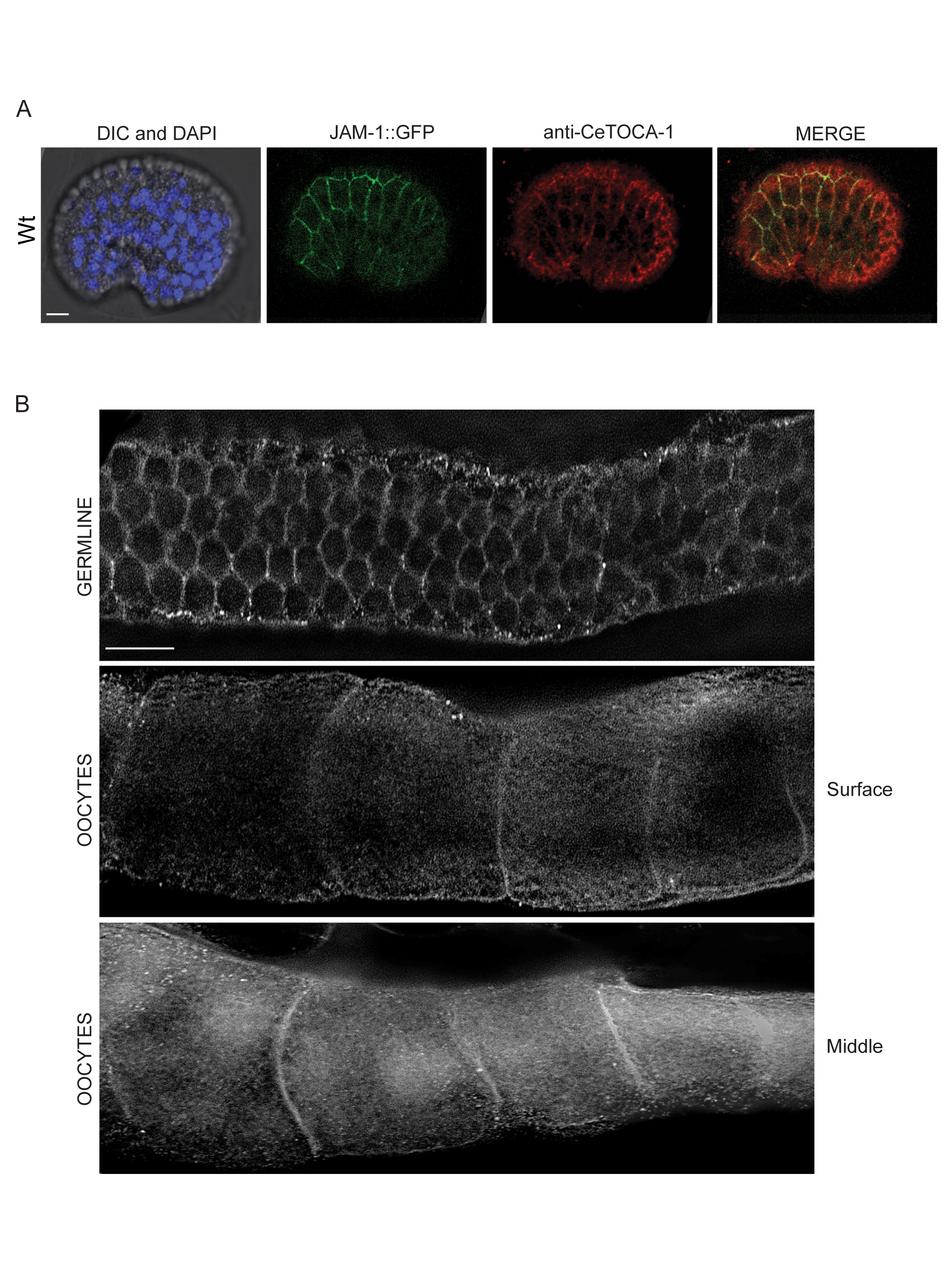

Supplement: Figure S2 — Toca localization at junction and in germline. (A) CeTOCA1 and AJM-1 partially colocalize at cell-cell junction. Confocal lateral view of Wt embryos expressing AJM-1::GFP at 1.5 fold stage. Embryos were fixed and stained with anti-CeTOCA-1 or processed for epifluorescence. Bar, 10 µm. (B) Germline and oocytes expression of CeTOCA-1. Germline and oocytes (surface and middle view) from Wt animal showing CeTOCA-1 expression. Gonads were dissected, fixed, and stained with anti-CeTOCA-1. Bar, 20 µm. Images were acquired with Axiovert 200 M microscope using MetaMorph and deconvoluted by AutoDeblur. (5.08 MB TIF) [file pgen.1000675.s002.tif]

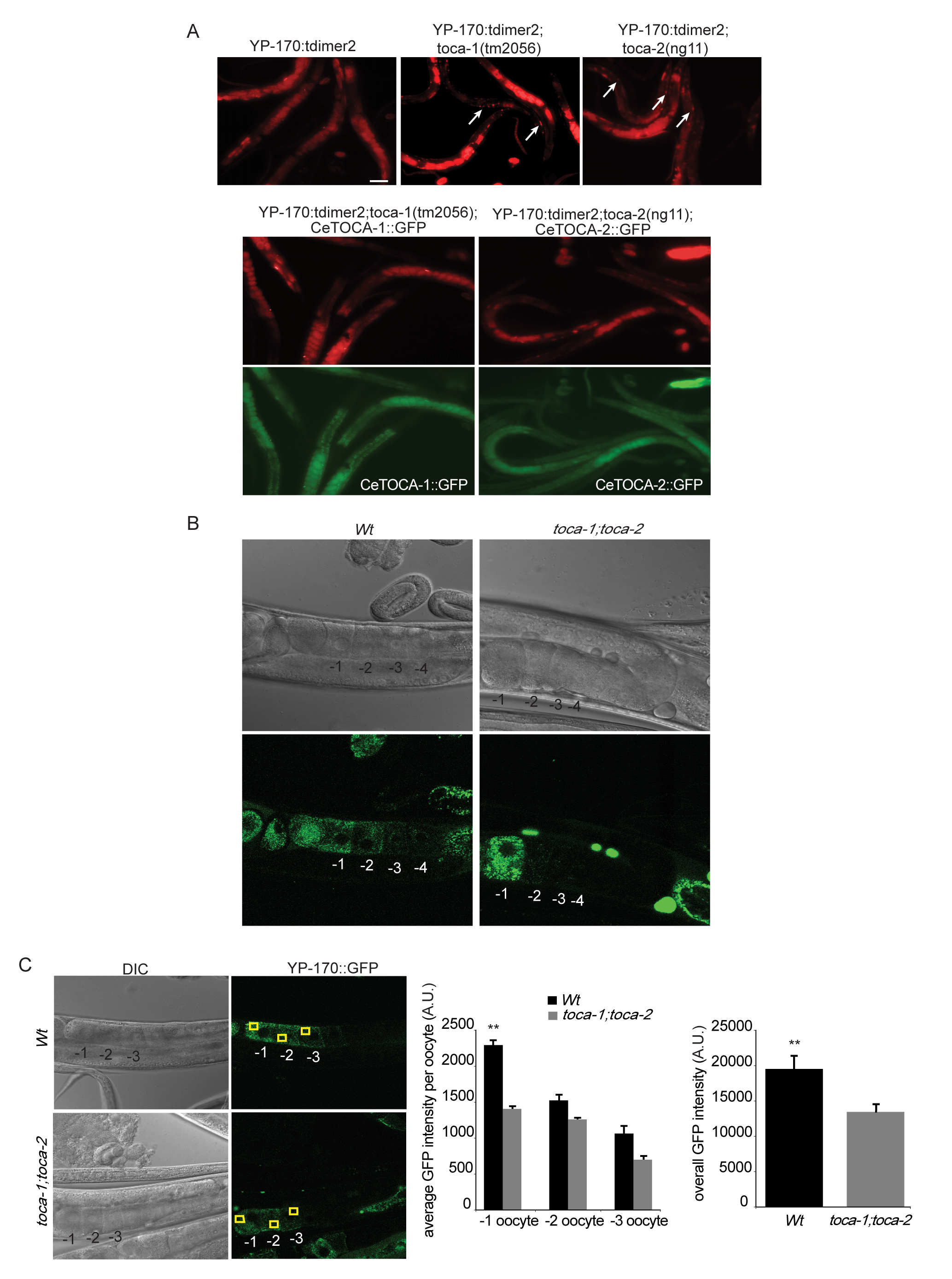

Supplement: Figure S3 — OCA proteins in yolk endocytosis. (A) pie-1::TOCA-1::GFP and pie-1::TOCA-2::GFP rescue the YP-170::tdimer2 accumulation in the body cavity of toca-1(tm2056) and toca-2(ng11) mutants. Localization of YP170::tdimer2 in synchronized young adult single toca-1 and toca-2 mutant worms and in pie-1::TOCA-1::GFP and pie-1::TOCA-2::GFP lines in their respective mutant background. Arrows indicate examples of YP-170::tdimer2 accumulation into the body cavity. Bar, 100 µm. (B) Double mutant toca-1;toca-2 display reduced YP-170::GFP endocytosis in the oocytes. Examples of the most represented categories of GFP-positive oocytes in Wt (3 oocytes, 80%) and toca-1;toca-2 mutant (1 oocyte, >85%) when comparing animals with the same number of oocytes in the gonad (see DIC images). The numbers −1, −2, −3, and −4 indicate the GFP positive oocytes from the more proximal to the more distal. (C) Double toca-1;toca-2 mutant has reduced YP-170::GFP in the oocytes. Left, quantification of YP-170::GFP into oocytes comparing Wt and toca-1;toca-2 with the same gonad category (3 GFP-positive oocytes). The numbers −1, −2, and −3 indicate the GFP positive oocytes from the more proximal to the more distal. YP-170::GFP fluorescent intensities (arbitrary units, A.U.) along selected (distance, pixel) area were quantified by ImageJ software (see Materials and Methods). Different areas within the three oocytes (e.g., yellow square) from at least 20 animals were analyzed. Right, graph showing the average GFP intensity per oocytes (left) or the overall GFP intensity (right) in Wt and toca-1;toca-2 mutant. Asterisks indicate P<0.0001 by two-tailed t-test. (2.93 MB TIF) [file pgen.1000675.s003.tif]

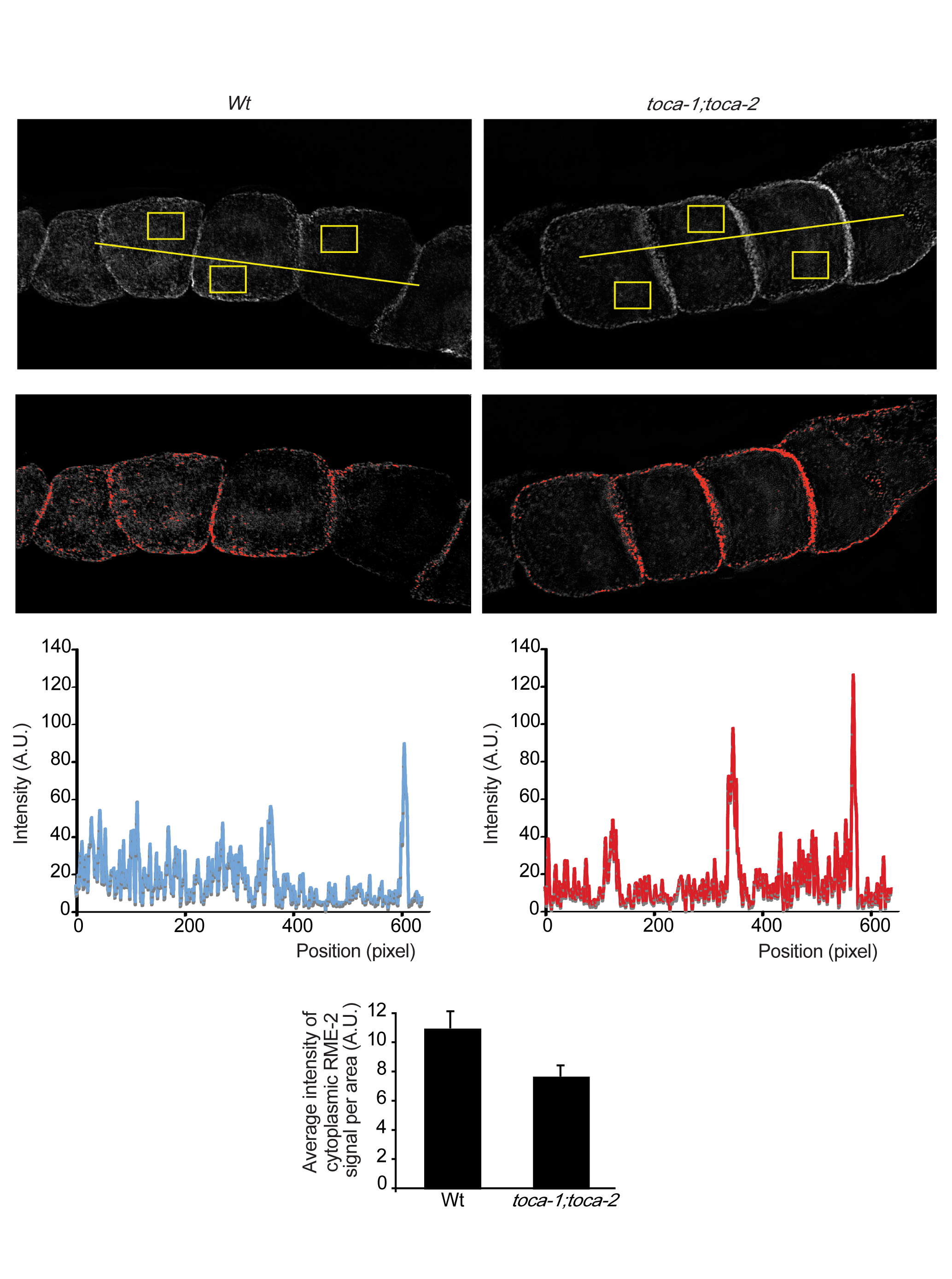

Supplement: Figure S4 — RME-2 levels in toca-1;toca-2 oocytes. RME-2, the yolk receptor, is correctly localized and enriched at the plasma membrane. RME-2::GFP fluorescent intensities (arbitrary units, A.U.) along selected (distance, pixel) areas and lines were quantified by ImageJ software (see Materials and Methods). Different areas from at least 20 Wt and toca-1;toca-2 animals were analyzed. The images in red represent a typical example of Wt and toca-1;toca-2 animals and were obtained by applying a threshold algorithm (ImageJ) to equalize and remove background staining and evidence pixel intensities values above threshold, which correspond to surface RME-2 signals. This procedure permits us to appreciate that the levels of cortical RME-2 are higher in toca-1;toca-2 animals with respect to Wt. Graph, the GFP intensity along the junctions (upper) and the average intensity of cytoplasmic RME-2 per area (bottom) is plotted for Wt and toca-1;toca-2. (1.68 MB TIF) [file pgen.1000675.s004.tif]

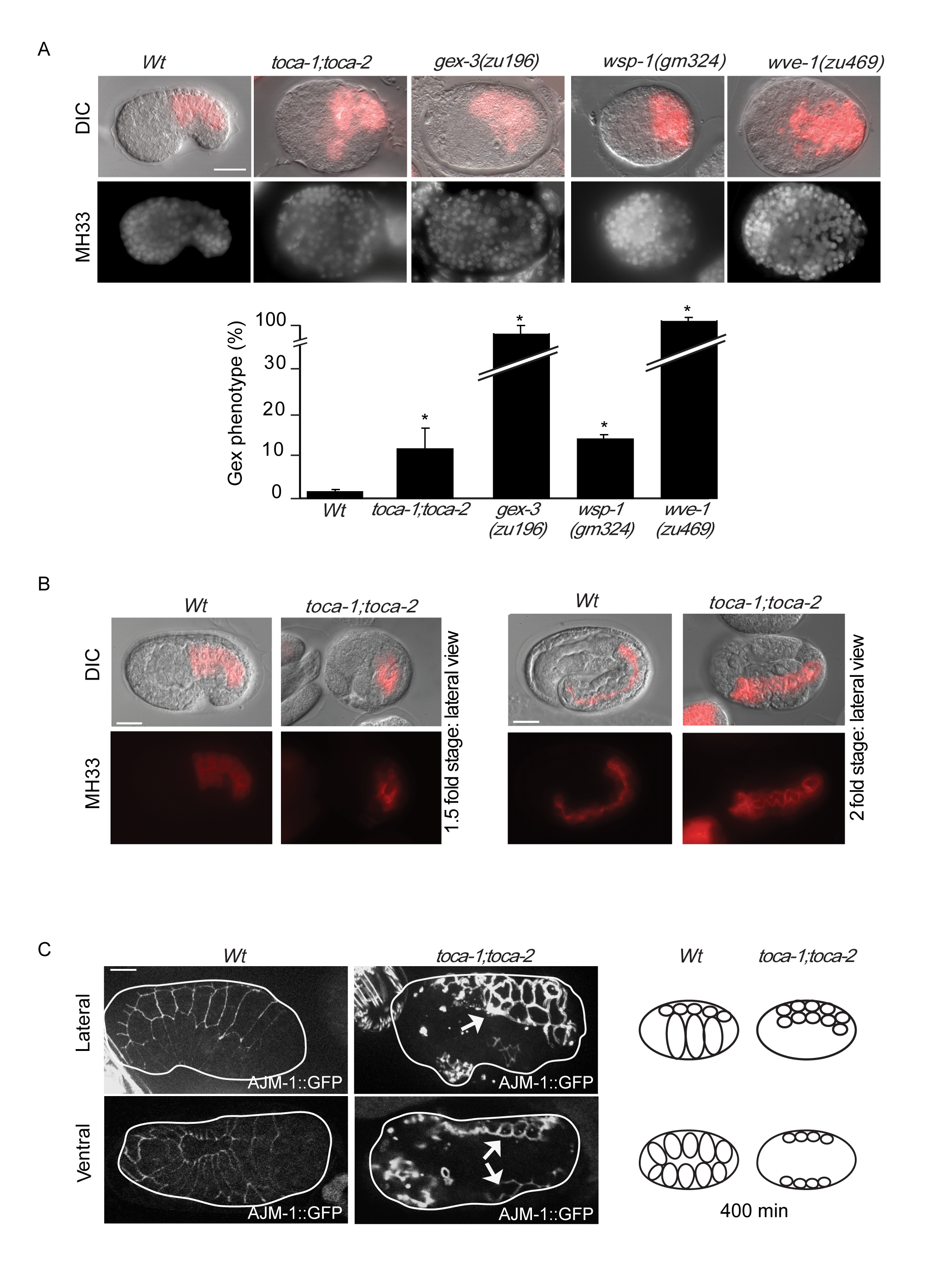

Supplement: Figure S5 — Intestinal morphology defects of toca-1;toca-2. toca-1 and toca-2 mutants display a Gex phenotype. (A) toca-1;toca-2 double mutant worm displays altered intestinal morphology during embryo development. Wt and the indicated mutant worms were fixed and stained with anti-MH33 antibody or DAPI to detect the intestinal cells and cell nuclei, respectively. Embryos die at 1.5 fold stage, just before elongation starts; DAPI shows that Wt and mutants have a similar number of nuclei, indicating a similar developmental stage. Bar, 10 µm. The percentage of gut-defective embryos of the various genotypes, quantified as described in Materials and Methods, is shown in the bottom graph. Please note that in the case of gex-3(zu196) we used a balanced heterozygous strain OX169 gex-3(zu196)/DnT1 in which only 25% of the progeny is homozygous for gex-3(zu196) according to Mendelian distribution. Nearly 100% of these homozygous gex-3(zu196) embryos display the morphogenetic intestinal defect as previously reported (Soto et al., 2002). Data are the mean±s.e.m. (n = 100) of at least three independent experiments. P<0.0001, two-tailed t-test is indicated by an asterisk. (B) Intestinal morphology of Wt and mutant embryo at different stages of development. Wt and toca-1;toca-2 mutant worms were fixed and stained with anti-MH33 antibody. All dying (∼12% of total embryos, Figure 5A) toca-1;toca-2 embryos are arrested at 1.5 fold stage, just before elongation starts (left); A significant fraction of toca-1;toca-2 embryos display altered intestinal morphology with enlargement of the intestinal lumen at the 2 fold stage with respect to Wt. Of note, at this stage it is easy to appreciate that MH33 display an apical distribution as previously reported (Patel et al., 2008) (right). Bar, 10 µm. (C) toca-1;toca-2 Gex (Gut on the exterior) embryos display an altered epidermal cell morphology typical of gex mutants. Left, lateral and ventral view of Wt and toca-1;toca-2 expressing AJM-1::GFP. Right, a sc [file pgen.1000675.s005.tif]

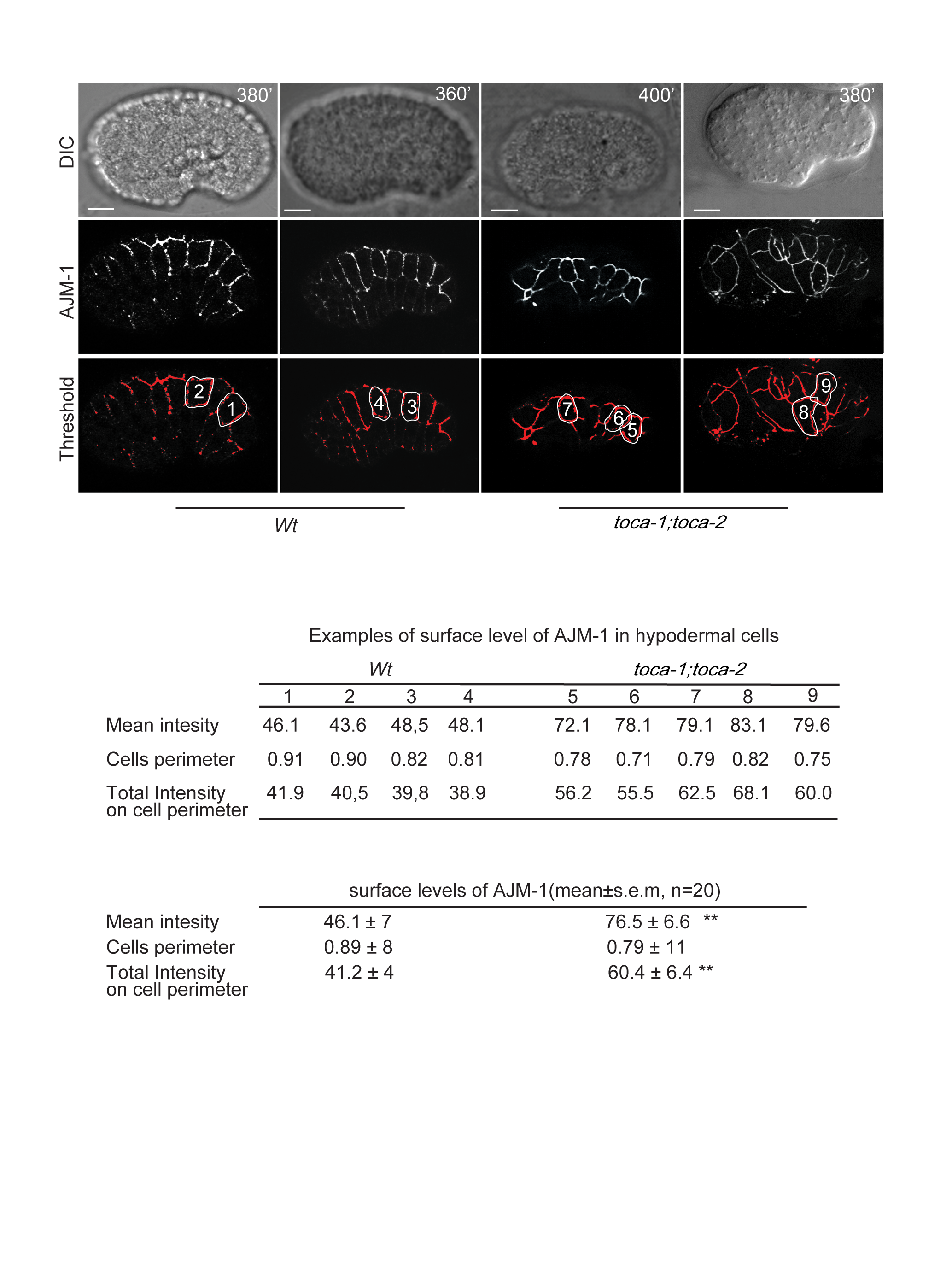

Supplement: Figure S6 — The surface levels of AJM-1 of toca-1;toca-2 mutant embryos are slightly higher than wt embryos. Quantification of AJM-1 along the cell perimeter of hypodermal cells in WT and toca-1;toca2 mutant embryos. Embryos at the two-cell stage were kept at 22° for 5 hours before fixation between 360 and 400 min, as indicated, when the “bean shape stage” was reached. Embryos were stained with anti-actin (not shown). The second and third raw images were used to determine the levels of F-actin at junctions as described in Figure 6B, or processed for epifluorescence or DIC. Images were captured with a Leica Microsystems confocal microscope using the HCX PL APO CS 63.0×1.40 OIL objective lens, and objective zoom (3.49×). Exposure time and gain setting were fixed as follows: (Dapi PMT1 (Photo Multiplier Tube) = 500, GFP-PMT2 = 583, Cy3-PMT3 = 550, PMT Trans (HV) = 298). Identical settings were used for all samples so that direct comparison of the signal intensities among the images of embryos of different genetic backgrounds was possible. The ImageJ threshold alogorithm (red channels) were then applied to eliminate cytoplasmic and background signals by placing “Regions of Interest” (ROI) over areas outside the junctional contour. We manually determined the cell perimeters and calculated mean intensities and perimeter length. The mean intensitiy values were then multiplied by the perimeter of each cell to obtain the total intensity along the cell perimeter, as exemplified in the boxed cells on the bottom raw (the line around the cells is meant to outline the chosen cell, not the actual perimeter). Examples of the values obtained for the outlined (white circles) cells are shown. We repeated the procedures to obtain statistically meaningful data that are expressed as mean±s.e.m (n>20 from at least 4 to 5 independent embryos of each genotype). The values of the total intensities along the cell perimeters were plotted as described in Figure 6A. Bar is 10 µm. The results indicate that t [file pgen.1000675.s006.tif]

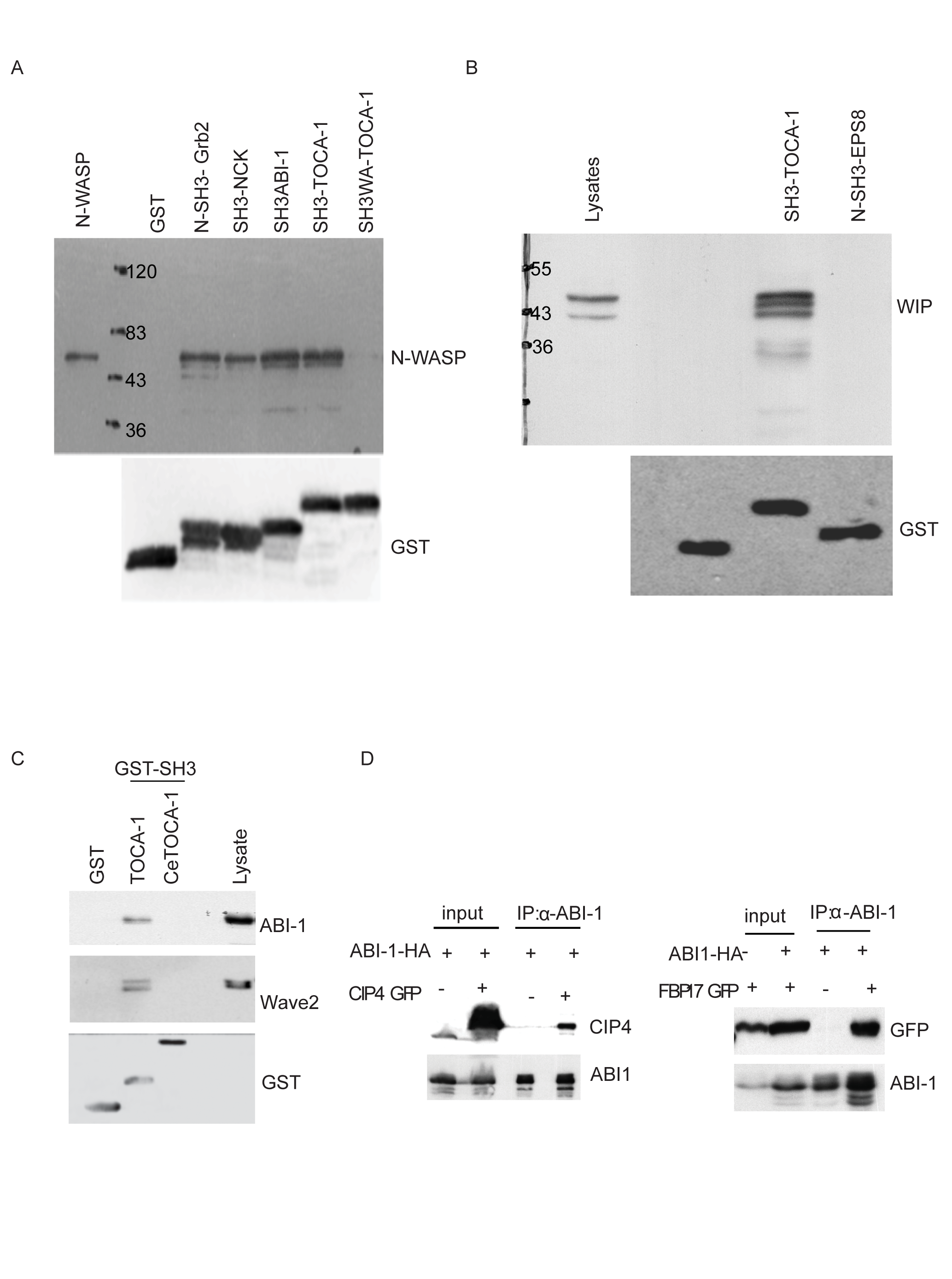

Supplement: Figure S7 — Biochemical interactions of Toca-1 protein in mammalian cells. (A,B) The SH3 domain of mammalian TOCA-1 binds to N-WASP and WIP. (A) Purified N-WASP was incubated with the indicated SH3-GST fusion proteins (10 µg). Input (1/10 of the total) and bound N-WASP and GST fusion proteins were detected with the antibodies indicated on the right. (B) Lysates (1 mg) from HeLa cells were incubated with the indicated SH3-GST fusion proteins (10 µg). Lysates (50 µg), bound material, and GST proteins were detected by immunoblotting with the indicated antibodies. (C) The SH3 domain of mammalian TOCA-1 binds to ABI1 and WAVE2. Lysates (2 mg) of HeLa cells were incubated with the SH3 domain indicated (SH3-GST) or GST alone as control (GST). Lysates (100 µg) and bound proteins were immunoblotted with the indicated antibodies. (D) CIP4 and FBP17 interact with ABI1. Lysates of HeLa cells expressing ABI1-HA alone or in combination with CIP4-GFP or FBP17-GFP were immunoprecipitated (IP) with anti-ABI1 antibody. Lysate and IP were immunoblotted with antibodies indicated on the right. (1.31 MB TIF) [file pgen.1000675.s007.tif]

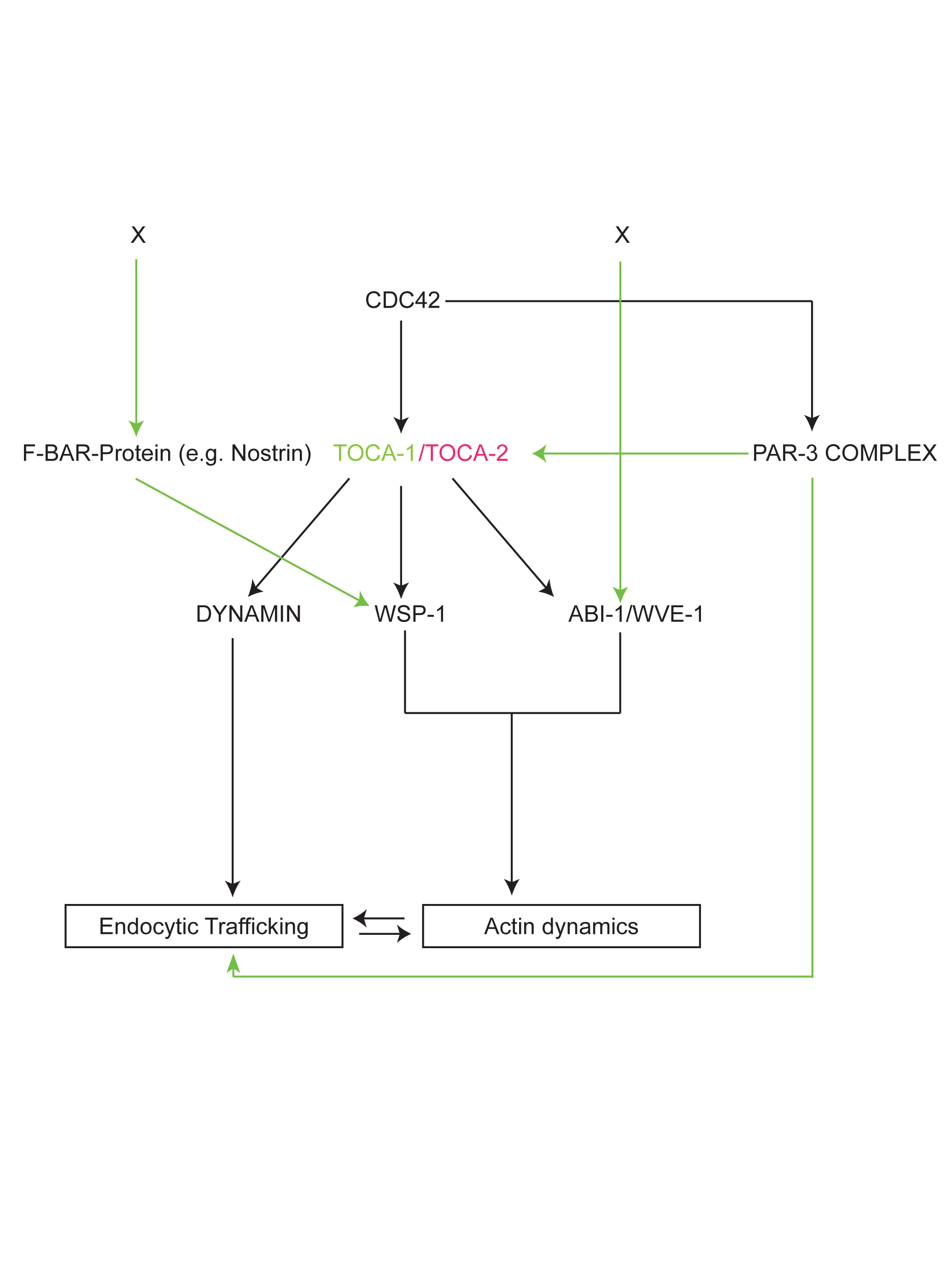

Supplement: Figure S8 — Working model of TOCA proteins signalling network in the regulation of membrane trafficking. Working model of TOCA proteins signalling network in the regulation of membrane trafficking. TOCA-1 and TOCA-2 may link nucleating promoting factors to the plasma membrane (not shown) via their F-BAR domain and integrate signalling pathways controlled by the small GTPases Cdc42. A Cdc42/WASP(WSP-1)/TOCA-1/2, in analogy to that demonstrated in mammalian (Itoh et al., 2005; Tsujita et al., 2006), may directly promote localized actin dynamics during early steps of Clathrin-mediated endocytosis (CME). The polarity complex PAR-3/PAR-6 whose activity is required for endocytic and recycling events downstream of Cdc42 (Balklava et al., 2007) may define an alternative branch of the pathway that may also converge in controlling the TOCA-1/2/WASP(WSP-1) axis. An unexpected contribution of the WAVE(WVE-1) axis in this process is evidenced by; i) the increased accumulation of YP170 after interference with WAVE (WVE-1) complex components; ii) the genetic interactions of these latter genes with toca-1/2; iii) the biochemical link between TOCA-1/2 and ABI1, which is conserved also in mammals. The WAVE (WVE-1) complex may function in later endocytic steps of CME since, at least, in mammals it does not localize to Clathrin-coats at the plasma Membrane (Benesch et al., 2005). The precise relation of the WAVE (WVE-1) complex with CDC42/TOCA-1/2 is unclear at present. However, TOCA-2 appears dominant with respect to TOCA-1. TOCA1/2 may also directly associate to Dynamin (Itoh et al., 2005; Tsujita et al., 2006), whose pinching activity is critical to promote vesicle scission. Dynamin- and actin-dependent activities may work in concert with TOCA-1/2 to promote tubule scission. Other F-BAR containing proteins, such as Nostrin may add further layers of complexity to this network, which may coordinate membrane tubulation and curvature sensing with the activity (WASP/WSP-1) (Itoh et al., 2005; Tsujit [file pgen.1000675.s008.tif]
