# Supplementary material for: Requirements for F-BAR Proteins TOCA-1 and TOCA-2 in Actin Dynamics and Membrane Trafficking during Caenorhabditis elegans Oocyte Growth and Embryonic Epidermal Morphogenesis
Source: PLoS Genet. 2009 Oct 2;5(10):e1000675. doi: 10.1371/journal.pgen.1000675 (PMC2744924; doi:10.1371/journal.pgen.1000675)
Supplement: Text S1 — Supplementary Materials and Methods. (0.04 MB DOC) [file pgen.1000675.s009.doc]

**Text S1**

**Supplementary Materials and Methods**

**Strains**

The following strains were used in this analysis: *toca-1(tm2056), toca-1(tm3334), toca-2(tm2088), toca-2(ng11), toca-1(tm2056);toca-2(ng11), toca-1(tm3334);toca-2(ng11), pie-1::TOCA-1::GFP;toca-1(tm2056), pie-1::TOCA-2::GFP;toca-2(ng11), wsp-1(gm324), toca-1(tm2056);toca-2(ng11);wsp-1(gm324), abi-1(ok640), gex-2(ok1603)/dpy-9 (out-cross 3), OX1 wve-1(zu469) unc-101(m1), OX169 gex-3(zu196)/DnT1, jcIs1(AJM-1::GFP), toca-1(tm2056);toca-2(ng11);jcIs1(AJM-1::GFP),*  *bIs1(VIT-2::GFP), toca-1(tm2056);bIs1(VIT-2::GFP), toca-1(tm3334);bIs1(VIT-2::GFP), toca-2(ng11);bIs1(VIT-2::GFP), toca-1(tm2056);toca-2(ng11);bIs1(VIT-2::GFP), pwIs116(RME-2::GFP) toca-1(tm2056);toca-2(ng11); pwIs116(RME-2::GFP), wsp-1(gm324);bIs1(VIT-2::GFP), abi-1(ok640);bIs1(VIT-2::GFP).* TOCA-1::GFP and TOCA-2::GFP were cloned under the *pie-1* promoter in pID-3.01B gateway vector and integrated transgenic lines in *toca-1(tm2056);unc-119(ed3)* and *toca-2(ng11);unc-119(ed3)* were obtained by the microparticle bombardment method (Praitis et al., 2001). *wve-1(zu469)* was discovered by Jim Priess. *tm2056*, *tm3334*, and *tm2088* were generated by Dr. Shohei Mitani in Tokyo Women's Medical University (Japan). *ng11* (549-2333 (1784 bp deletion)) was generated using the random mutagenesis method with TMP/UV combined with gene-specific primer sets. All *tocas* alleles were outcrossed 4 times with N2 before phenotypic analysis, and the loci were sequenced to confirm the deletion annotated in Wormbase. Some of the strains used in this study were provided by the *Caenorabditis* Genetic centre (supported by National Institute of Health)*. pwIs116(RME-2::GFP)* was generated by GB.

**RNAi interference**

RNA interference against *wve-1, gex-2, gex-3, cdc-42, chc-1* was obtained by feeding experiments using clones of the RNAi feeding library constructed by J. Ahringer’ lab. The feeding RNAi was carried out as following: saturated overnight cultures were diluted 1:40 in LB with Ampicillin 100µg/ml and tetracycline 15 µg/ml and grown until the OD600 was close to 0.4. 0.4 mM IPTG was added and bacteria were grown for 4 hours. Bacteria were then resuspended in 1/10 of the starting volume and seeded on plates supplied with Ampicillin 100µg/ml, tetracycline 15 µg/ml and IPTG 30 mM. *C. elegans* synchronous L1 larvae were fed either with control HT115 *E. Coli* containing the empty vector L4440 or the L4440 plasmid carrying the gene of interest. Worms were grown for 2 generations on RNAi expressing bacteria in the N2 or YP-170::GFP background before phenotypic analysis. As a control, the embryonic lethality caused by RNAi-mediated interference of *rad-51*, an unrelated essential gene involved in DNA repair, was not enhanced in the *toca-1:toca-2* double mutant.

***C. elegans* lysates for biochemistry**

Worms were suspended in either a Triton X100-based Lysis Buffer (200 mM NaCl, 20mM Tris pH 7.4, 10% Glycerol, 1% Triton and protease inhibitor cocktail — Roche) or RIPA buffer (15 mM Hepes pH 7.2, 140 mM NaCl, 3 mM MgCl_2_, 1 mM EDTA, 0.5% sodium deoxycholate, 1% NP-40, 2% SDS, protease inhibitor cocktail -Roche). Acid-washed glass beads (Sigma,) and FastPrep®-24 sample preparation system (MP Biomedicals) were used to homogenize worms (4.0 m/s for 45 seconds at RT). Lysates were spun at full speed at 4º C. The amount of total protein was determined and supernatants were flash frozen in liquid nitrogen to be used for immunoblotting analysis.

**Pulldown, Coimmunoprecipitation and Biochemical assays.**

Cells were lysed in a buffer containing 1% Triton X-100 (Pierce, Rockford, IL), 50 mM HEPES, pH 7.5, 150 mM NaCl, 10% glycerol, 1.5 mM MgCl2, and 5 mM EGTA, adding the Calbiochem protease inhibitor cocktail (ID-539134; La Jolla, CA), and lysates were left rocking on ice for 15 min. For pulldown experiments, 20 µg of GST-fusion proteins attached to the glutathione-Sepharose beads were incubated in the presence of 1 mg of total cell lysate for 1 h at 4°C rocking. After three washes in lysis buffer, bound proteins were resolved by SDS-PAGE and visualized with the appropriate antibodies. For coimmunoprecipitation studies, cells were lysed and cell lysates were used immediately, without freeze/thawing. Immunoprecipitations and coimmunoprecipitation experiments were performed for 1 h in the presence of the appropriate antibody, and immune complexes were recovered by adsorption to protein G-Sepharose beads (Zymed, South San Francisco, CA). After three washes in lysis buffer, bound proteins were resolved by SDS-PAGE and visualized with the appropriate antibodies.

When Rac, Rho, Cdc42 GTPγ-S or GDPγ-S was used, *in vitro* binding was performed in nucleotide-loading buffer to preserve Rac–Cdc42 loading (Scita et al., 1999).

**Nematode immunostaining and microscopy**

For nematode staining, embryos were attached to poly-l-lysine slides and permeabilized by freeze cracking after at least 5 min on dry ice. The samples were fixed in fixing solution (75% Methanol, 4% Formaldehyde, 0.5x PBS, 0.1 mM EGTA, H2O to final volume) at room temperature for 15 min, blocked in PBSBT (PBS with 0.25% Tween 20 and 1% BSA) for 30 min, then incubated in primary antibodies diluted 1:20 in PBSBT at 4 °C overnight in humid chamber. Fluorescence-coupled secondary antibodies diluted 1:200 were added overnight at 4 °C in PBST (PBS with 0.2% Tween 20) in humid chamber. For Phalloidin staining, embryos were attached to poly-l-lysine slides, permeabilized by freeze cracking as above, and fixated in 4% paraformaldehyde at room temperature for 10 min. Embryos were incubated in rhodamine-conjugated Phalloidin diluted in PBST at room temperature for 1 h. All slides were mounted in 1x PBS containing 70% glycerol and 0.4 mM DAPI. All antibody-staining images were acquired on a Zeiss Axioplan 2 plus microscope with an Axiocam HRc, except if otherwise indicated in the legends of the Figures. Quantitation of antibody staining was performed using ImageJ software including the rectangular selection and mean measure tools to measure average fluorescence of equal areas.

**Total eggs laid quantification and embryonic lethality**

**Total eggs laid**. Single L4 larvae (at least 15) were plated in small agar plates and moved to a new plate every 24 hours. Eggs laid were counted every day until the adult stopped producing eggs.

**Embryonic lethality**. Single L4 larvae (at least 15) were plated in small agar plates and moved to a new plate every 24 hours. Eggs were counted and kept at 20C. Eggs unable to hatch were counted after 24 hours.

**Endocytosis of YP170::GFP.** *toca-1* and *toca-2* single and double mutants were crossed to *bIs1(VIT-2::GFP)* transgenic line and then hypochlorite-synchronised young adults were analysed. Worms (at least 100) were analyzed by Zeiss Axioplan 2 plus microscope with a Axiocam HRc, unless otherwise indicated.

**Immunofluorescence**

Cells seeded on gelatine for 24 h, were processed for epifluorescence or indirect immunofluorescence microscopy. Briefly, cells fixed in 4% paraformaldehyde and permeabilized in 0.1% Triton X-100 were incubated with the indicated primary antibody followed by the appropriate secondary antibody. Confocal Microscopy was performed on a Leica TCS SP2 AOBS confocal microscope equipped with blue (Argon, 488 nm), yellow (561 nm Solid State Laser), and red (633 nm HeNe Laser) excitation laser lines. A 633/1.4 NA oil-immersion objective (HCX PL APO 63X Lbd Bl, Leica Microsystems) was employed for analysis. Image acquisition conditions were set to remove channel crosstalk, optimizing spectral detection bands and scanning modalities. Leica Confocal Software and ImageJ were used for data analysis.

**Statistical analysis**

In all graphs reported are the mean values ± S.D. of three experiments performed in triplicate or the percentage ± S.E.M, except if otherwise indicated in the legends to the Figures. In all cases statistical analysis was performed using an unpaired two-tail t-test. Asterisks (*) indicate that the differences observed are statistically significant.

**Supplementary References**

Balklava, Z., Pant, S., Fares, H., and Grant, B.D. (2007). Genome-wide analysis identifies a general requirement for polarity proteins in endocytic traffic. Nat Cell Biol *9*, 1066-1073.

Benesch, S., Polo, S., Lai, F.P., Anderson, K.I., Stradal, T.E., Wehland, J., and Rottner, K. (2005). N-WASP deficiency impairs EGF internalization and actin assembly at clathrin-coated pits. J Cell Sci *118*, 3103-3115.

Itoh, T., Erdmann, K.S., Roux, A., Habermann, B., Werner, H., and De Camilli, P. (2005). Dynamin and the actin cytoskeleton cooperatively regulate plasma membrane invagination by BAR and F-BAR proteins. Dev Cell *9*, 791-804.

Patel, F.B., Bernadskaya, Y.Y., Chen, E., Jobanputra, A., Pooladi, Z., Freeman, K.L., Gally, C., Mohler, W.A., and Soto, M.C. (2008). The WAVE/SCAR complex promotes polarized cell movements and actin enrichment in epithelia during C. elegans embryogenesis. Dev Biol.

Praitis, V., Casey, E., Collar, D., and Austin, J. (2001). Creation of low-copy integrated transgenic lines in Caenorhabditis elegans. Genetics *157*, 1217-1226.

Scita, G., Nordstrom, J., Carbone, R., Tenca, P., Giardina, G., Gutkind, S., Bjarnegard, M., Betsholtz, C., and Di Fiore, P.P. (1999). EPS8 and E3B1 transduce signals from Ras to Rac. Nature *401*, 290-293.

Soto, M.C., Qadota, H., Kasuya, K., Inoue, M., Tsuboi, D., Mello, C.C., and Kaibuchi, K. (2002). The GEX-2 and GEX-3 proteins are required for tissue morphogenesis and cell migrations in C. elegans. Genes Dev *16*, 620-632.

Tsujita, K., Suetsugu, S., Sasaki, N., Furutani, M., Oikawa, T., and Takenawa, T. (2006). Coordination between the actin cytoskeleton and membrane deformation by a novel membrane tubulation domain of PCH proteins is involved in endocytosis. J Cell Biol *172*, 269-279.
